# Cerebrospinal fluid markers link to synaptic plasticity responses and Alzheimer’s disease genetic pathways

**DOI:** 10.1186/s13024-025-00899-w

**Published:** 2025-10-13

**Authors:** Bjørn-Eivind Kirsebom, Johanna Nilsson, Ellen Vromen, Peter Mikael Arnesen, Atle Bjørnerud, Ann Brinkmalm, Geir Bråthen, Gøril Rolfseng Grøntvedt, Jonas Jarholm, Kaja Nordengen, Lene Pålhaugen, Per Selnes, Nikias Siafarikas, Ragnhild Eide Skogseth, Sandra Tecelão, Knut Waterloo, Panpan You, Henrik Zetterberg, Dag Aarsland, Betty Tijms, Pieter Jelle Visser, Kaj Blennow, Tormod Fladby

**Affiliations:** 1 Department of Neurology, University Hospital of North Norway, Tromsø, 9038 Norway; 2 Department of Psychology, Faculty of Health Sciences, UiT, The Arctic University of Norway, Tromsø, Norway; 3 Department of Neurology, Akershus University Hospital, Lørenskog, Norway; 4 Department of Psychiatry and Neurochemistry, Institute of Neuroscience and Physiology, The Sahlgrenska Academy, University of Gothenburg, Gothenburg, Sweden; 5 Alzheimer Center Amsterdam, Neurology, Vrije Universiteit Amsterdam, Amsterdam UMC Location VUmc, Amsterdam, The Netherlands; 6 Amsterdam Neuroscience, Neurodegeneration, Amsterdam, The Netherlands; 7 Division of Radiology and Nuclear Medicine, Oslo University Hospital, Oslo, Norway; 8 Clinical Neurochemistry Laboratory, Sahlgrenska University Hospital, Mölndal, Sweden; 9 Department of Neurology and Clinical Neurophysiology, University Hospital of Trondheim, Trondheim, Norway; 10 Department of Neuromedicine and Movement Science, Faculty of Medicine and Health Sciences, Norwegian University of Science and Technology, Trondheim, Norway; 11 Department of Neurology, Oslo University Hospital, Oslo, Norway; 12 Department of Geriatric Psychiatry, Akershus University Hospital, Lørenskog, Norway; 13 Department of Geriatric Medicine, Neuro-SysMed, Haraldsplass Deaconess Hospital, Bergen, Norway; 14 Institute of Clinical Science, Department of Medicine, University of Bergen, Bergen, Norway; 15 Department of Neurodegenerative Disease, University College London Institute of Neurology, London, UK; 16 UK Dementia Research Institute at University College London, London, UK; 17 Hong Kong Centre for Neurodegenerative Diseases, Hong Kong, China; 18 Wisconsin Alzheimer’s Disease Research Center, University of Wisconsin School of Medicine and Public Health, University of Wisconsin-Madison, Madison, WI USA; 19 King’s College London, Institute of Psychiatry, Psychology and Neuroscience, London, UK; 20 Centre for Age-Related Medicine, Stavanger University Hospital, Stavanger, Norway; 21 Paris Brain Institute, ICM, Pitié-Salpêtrière Hospital, Sorbonne University, Paris, France; 22 Neurodegenerative Disorder Research Center, Division of Life Sciences and Medicine, and Department of Neurology, Institute on Aging and Brain Disorders, University of Science and Technology of China and First Affiliated Hospital of USTC, Hefei, P.R. China; 23 Institute of Clinical Medicine, Campus Ahus, University of Oslo, Lørenskog, Norway

**Keywords:** Alzheimer’s disease, Memory, Cognition, Synaptic loss, Neurodegeneration

## Abstract

**Background:**

Synapse loss is linked to cognitive symptoms in Alzheimer’s Disease (AD) and Cerebrospinal fluid (CSF) synaptic biomarkers may clarify disease heterogeneity and disease mechanisms for progression beyond amyloid (Aβ) and tau pathologies, potentially revealing new drug targets.

**Methods:**

We used a mass-spectrometry panel of 17 synaptic biomarkers including neuronal pentraxins (NPTXs) linked to glutamatergic signaling, and 14-3-3 proteins linked to tau-pathology and synaptic plasticity. Synapse markers were evaluated in two independent cohorts: Dementia Disease Initiation (DDI) (*n* = 346) and Amsterdam Dementia Cohort (n = *397*), both with cognitive assessments up to 10 years. We used linear regression to compare synapse marker differences between CSF-determined Aβ + cognitively normal (CN) and Mild Cognitive Impairment (MCI) groups, with or without CSF tau pathology (Tau+/-), relative to CN Aβ-/Tau- controls; and associations between synapse markers and medial temporal lobe (MTL) MRI volumetrics in the DDI cohort and with verbal memory in both cohorts. A funneling procedure identified proteins related to Aβ/Tau pathology and memory impairment in both cohorts, which were used to evaluate relations to Aβ/Tau biological progression in the DDI cohort and memory decline in both cohorts. Finally, we explored genetic pathways associated with these synaptic proteins.

**Results:**

In both cohorts, most markers were elevated in Aβ+/Tau + cases compared to controls, particularly 14-3-3ζ/δ. Several proteins were reduced in Aβ+/Tau- cases, especially NPTX-2, while 14-3-3ζ/δ remained elevated. However, the increase in e.g. 14-3-3ζ/δ and reduction in e.g. NPTX2 were more pronounced in patients with MCI than CN cases regardless of tau-pathology, corresponding to verbal memory impairment and MTL atrophy. Elevated baseline 14-3-3ζ/δ and rab GDP Dissociation Inhibitor Alpha (GDI-1) associated with future progression from Aβ+/Tau- to Aβ+/Tau+. Significant associations (all *p* < 0.001) were found between 14-3-3 protein genes (*YWHAZ*, *YWHAE*) and pathways linked to AD, including the p38 MAPK, IGF, PIK3/AKT and between *GDI1* and p38 MAPK upstream pathway (*p* < 0.05) all connected to synaptic plasticity. Correspondingly, a robust 14-3-3ζ/δ association with future memory decline was observed in both cohorts.

**Conclusions:**

Reduced markers for excitatory signaling in Aβ+/Tau- and increased synaptic plasticity markers in Aβ+/Tau + cases suggest differential but linked processes underlying disease progression and resilience in the groups.

**Supplementary Information:**

The online version contains supplementary material available at 10.1186/s13024-025-00899-w.

## Background

The increasing prevalence of persons with dementia poses a major global challenge, as projections indicate it will affect over 100 million people by 2050, with Alzheimer’s Disease (AD) being the most common cause [1]. Given the insufficient efficacy of available therapies [2, 3] there is a need for improved understanding for pathogenetic mechanisms and additional treatment targets.

Most AD patients show a characteristic sequence of cognitive impairment, with initial loss of verbal recall and other memory functions corresponding to patterns of brain atrophy with early loss of entorhinal and hippocampal tissues, with synapse loss as a key factor [4–7]. Recent diagnostic advances in AD have enabled high diagnostic accuracy through the integration of positron emission tomography (PET) targeting β-amyloid (Aβ) and tau pathologies, along with blood and cerebrospinal fluid (CSF) analyses of Aβ42/40 ratio and the phosphorylated tau (P-tau) epitopes, which serve as core AD biomarkers [8–11]. Lately, tracers targeting synaptic proteins such as Synaptic Vesicle Glycoprotein 2 A (SV2A) have become available, showing biomarker evidence of Aβ pathology-related synapse loss in the entorhinal cortex (ERC) and the hippocampus, putatively connected to early AD ERC pathology affecting excitatory afferents to the hippocampus [12]. Thus, synapse dysfunction and loss are well documented features of AD and closely linked to cognitive impairment [13, 14]. Though markers for synaptic pathology are not included among core AD diagnostic criteria, synaptic markers may refine predictions of cognitive decline and fortify links between putative mechanistic causes for AD and loss of cognitive function. Understanding the interplay between synaptic, amyloid and tau-related pathways may expand our comprehension of mechanisms driving disease progression and resilience, and potentially unveil new drug targets.

The prevalent understanding of AD progression based on the amyloid cascade hypothesis posits Aβ pathology as an upstream driver, inducing tau pathology, synaptic dysfunction, inflammation and other downstream pathologies [15, 16]. In this scenario, increased tau pathology and widespread neuronal loss later in the disease course are associated with increases in CSF synapse marker concentrations [17]. While elevated CSF total-tau is generally considered a marker of neurodegeneration, it is coupled to glial activation and may also reflect abnormal plasticity processes occurring in AD [18, 19]. Indeed, experimental deafferentation is well known to induce CNS plasticity responses, e.g. as described in the hippocampus following perforant path lesions (connecting the ERC and dentate gyrus) [20]. These results are consistent with glutamatergic loss seen in post-mortem studies, and results on AD animal models showing excitatory receptor disruption in the trisynaptic circuit including the dentate gyrus and may suggest a role for neuronal pentraxins (NPTXs) [21–24]. 

Secreted NPTXs and the neuronal pentraxin receptor (NPTXR) have been implicated in excitatory synaptogenesis, hippocampal signaling and synapse loss, in particular clustering of glutamate receptors and mediating the effects of Aβ [21, 25]. 14-3-3 proteins are markers of neurodegeneration known to be upregulated in Alzheimer’s disease [26]. They are ubiquitously expressed, but enriched at synapses with key functions in glutamatergic signaling and synaptic plasticity, and show phosphorylation-dependent activity [27]. They also enhance kinase-dependent processes including tau phosphorylation and aggregation and co-localize with tau in neurofibrillary tangles [28–30]. The rab GDP dissociation inhibitor alpha (GDI-1) regulates endocytotic and endolysosomal vesicle cycles [31]. Synaptic proteins like neurogranin and GAP43 have been explored as potential fluid biomarkers for Alzheimer’s disease [32]. 

To further explore synaptic dysfunction in Alzheimer’s disease, we recently developed a mass spectrometric assay to quantify synaptic protein levels in the CSF [33]. The method includes a panel of 16 synaptic proteins encompassing neurogranin, the activating protein 2 subunit complex beta (AP2B1), complexin-2, rab GDP dissociation inhibitor alpha (GDI-1), and several members of the protein families of the 14-3-3s, syntaxins, synucleins, granins, Neurosecretory Protein VGF (VGF), and NPTX1, NPTX2 and NPTXR. These protein concentrations have previously been shown to be increased (β-synuclein, γ-synuclein, neurogranin and 14-3-3ζ/δ proteins), decreased (neuronal pentraxins, granins, and VGF), and unchanged (complexin-2, GDI-1, and AP2B1) in the CSF of preclinical, prodromal and AD dementia cases compared to controls [33–35]. 

By utilizing the panel method as well as measuring growth associated protein 43 (GAP-43) with an ELISA, and utilizing untargeted tandem mass tag mass spectrometry (TMT-MS) proteomics in an independent cohort, we cover an array of synaptic proteins with diverse functions and localizations in two large cohorts comprising cognitively normal (CN) and mild cognitive impairment (MCI) cases, staged according to the significant CSF determined Aβ and tau pathologies. This allows for a comprehensive view of synaptic dysfunction related to differential expression of core pathologies in AD along the predementia continuum. The panel approach also allows for study of pathway-associations of pertinent involved genes.

Thus, our main objective is to study key synaptic proteins in early predementia AD cases, both with or without significant CSF determined Aβ and tau pathology in two large and well characterized independent cohorts. While numerous studies demonstrate associations between synapse markers and disease, some of the markers may play a bystander role. We hypothesize that using a panel approach combined with multimodal techniques and longitudinal follow-up will reveal the markers and mechanisms critically involved in disease progression. Importantly, our study addresses the role of Aβ and tau pathology across both CN and MCI cases. By doing so, we offer a broader perspective on synaptic changes in early AD than previously reported, capturing both asymptomatic and symptomatic stages within the disease continuum. Specifically, we ask which proteins are most strongly connected to core AD pathologies at both asymptomatic and symptomatic stages, whether these proteins are associated with atrophy in critical medial temporal lobe subregions such as the ERC and hippocampus, how they relate to cognitive impairment, whether they associate with disease progression or resilience and whether gene expression patterns for significantly altered proteins are connected to distinct disease-related pathways. Understanding pathway activations and expression patterns of these markers will inform novel and attractive targets for precision intervention in Alzheimer’s disease.

## Methods

### The Dementia Disease Initiation cohort

The Norwegian multi-center study Dementia Disease Initiation (DDI) cohort comprises individuals aged between 40 and 80 years recruited from memory clinics and advertisements in local news media [36]. Subjective Cognitive Decline (SCD) was classified according to the SCD-I framework, which requires normal performance on neuropsychological tests while experiencing a subjective decline in any cognitive domain [37]. Mild Cognitive Impairment (MCI) was classified according to the National Institute on Aging and Alzheimer’s Association (NIA-AA) criteria, which require the presence of subjective cognitive impairment or decline in combination with lower performance than expected in one or more cognitive domains, yet preserved independence in functional ability and not fulfilling the criteria of dementia [38]. Cases recruited as healthy controls reported no subjective cognitive decline and were recruited from advertisements, spouses of patients with dementia/cognitive disorder, and patients who received lumbar puncture for orthopedic surgery. The presence of cognitive impairment was determined when results were 1.5 SD below the normative mean within one or more cognitive domains, including delayed memory recall (Consortium to Establish a Registry for Alzheimer’s Disease (CERAD) word list test) [39, 40], executive function (Trail Making Test part B (TMT-B)) [41, 42], language/verbal fluency (Controlled Oral Word Association Test (COWAT)) [43, 44] and visuoperceptual ability (Visual Object and Space Perception Battery (VOSP) silhouettes) [45, 46]. 

### The Amsterdam Dementia Cohort

The Amsterdam Dementia Cohort (ADC) is comprised of individuals who visited the Alzheimer Center of the Amsterdam UMC, location VU University Medical Center (VUmc) in Amsterdam, which is a tertiary memory clinic [47]. Individuals referred to the Alzheimer Center Amsterdam receive standardized diagnostic work-up, including neurological investigation by a neurologist, neuropsychological testing, assessment of vital functions and CSF. Diagnoses were made based on international consensus criteria [48–50] during a weekly multidisciplinary meeting. Individuals were included when they had CSF proteomics measurements available, from which the synaptic proteins were selected, and when they had a diagnosis of subjective cognitive decline (SCD, considered as cognitively normal) or mild cognitive impairment due to AD.

### Cerebrospinal fluid core AD markers in DDI and ADC cohorts

In DDI, lumbar punctures were performed between 9 and 12 AM, and CSF samples were collected in sterile polypropylene tubes and centrifuged. Commercial enzyme-linked immunosorbent assays (ELISAs) from Fujirebio, Ghent, Belgium (Innotest) based on monoclonal antibodies to determine CSF concentrations of total tau (t-tau, hTAU-Ag kits) and phosphorylated tau (PhosphoTAU-181p kit). The QuickPlex SQ 120 system from Meso Scale Discovery (MSD, MD, USA) was used to measure Aβ_1−42_, Aβ_1−40_ in a multiplex setup using V-plex Ab Peptide Panel 1 (6E10) kit (K15200E-1). The cut-off values for CSF t-tau (≥ 378). and p-tau abnormality (≥ 66.5) were applied according to unpublished cut-offs derived from receiver operating curve (ROC) analyses (Aβ- healthy controls versus Aβ + MCI/Dementia) within the DDI cohort. The cut-off for CSF Aβ_42/40_ ratio at ≤ 0.077 was determined following ROC analysis using visual read of [18 F]-flutemetamol PET scans as the standard of truth [51]. In ADC, a lumbar puncture was performed to collect CSF, preferably using a 25-gauge needle and collected in polypropylene tubes. CSF was processed according to international criteria [52–54]. Concentrations of Aβ_1−42_ (Amyloid β 1–42 kit), p-tau (PhosphoTAU-181p kit) and t-tau (hTAU-Ag kit) were determined using ELISA assays from Innotest (Fujirebio, formerly Innogenetics, Ghent, Belgium). The previously published cutoff of drift-corrected < 813 pg/ml was used to determine Aβ_1−42_ abnormality [54], > 52 pg/ml for p-tau abnormality and > 375 pg/ml for t-tau abnormality [55]. 

### Study design

We initially used the A/T/N classification scheme [56] for hallmark AD biomarkers in both DDI and ADC cohorts to determine the presence of amyloid plaques (A, CSF Aβ_42/40_ ratio in DDI, Aβ_1−42_ in ADC), neurofibrillary tangles (T, CSF p-tau181) and evidence of neurodegeneration (N, CSF t-tau). As p-tau181 and t-tau are closely correlated in AD [57], we opted to simplify our groups by combining T and N status as “Tau+” (T + and/or N+) or “Tau-” (T- and N-) providing a scheme we have termed Aβ+/- / Tau+/-. In both cohorts we selected three groups: (1) Cognitively normal (Controls or SCD) with normal biomarkers (CN Aβ-/Tau-), (2) CN or MCI cases with amyloid pathology, but normal p-tau and t-tau (Aβ+/Tau-) and (3) CN or MCI cases with both amyloid pathology and at least one pathological tau marker (p-tau and/or t-tau) (Aβ+/Tau+). The Aβ + groups were further grouped according to CN/MCI status. The DDI sample comprised a total of 346 participants (CN Aβ-/Tau-, *n* = 154; CN Aβ+/Tau-, *n* = 20; MCI Aβ+/Tau-, *n* = 25; CN Aβ+/Tau+, *n* = 40; MCI Aβ+/Tau+, *n* = 107). Please note that 8 (Aβ+/Tau-, *n* = 2; Aβ+/Tau+, *n* = 6) of participants (11.3%) recruited as controls had one or more impaired cognitive scores, possibly consistent with MCI and had pathological AD biomarkers. These participants were thus classified as MCI. In addition, 274 (79%) had available repeated longitudinal cognitive measurements in DDI (between 2 and 11 repeated measurements between 0.52 and 9.67 years from baseline (*M* = 3.47, *SD* = 1.70, total observations = 789). The ADC sample comprised a total of 397 participants (CN Aβ-/Tau-, *n* = 187; CN Aβ+/Tau-, *n* = 60; MCI Aβ+/Tau-, *n* = 35; CN Aβ+/Tau+, *n* = 47; MCI Aβ+/Tau+, *n* = 68). Of these, 333 (84%) had repeated cognitive test measurements available (between 2 and 9 repeated measurements between 0.45 and 9.87 years from baseline (*M* = 2.79, *SD* = 1.85, total observations = 1195). Please see Table 1 for detailed overview of baseline DDI and ADC characteristics. To assess associations with biological disease progression, additional cases from the DDI cohort was sourced: controls that remained both CN and Aβ-/Tau- over time (*n* = 58), cases progressed from Aβ-/Tau- to Aβ+/Tau- *n* = 23) and cases progressed from Aβ+/Tau- to Aβ+/Tau+ (*n* = 8). No cases with biological progression were available from the ADC cohort.

**Table 1:** Between-group comparisons of demographics, *APOE-ε4* carrier status and diagnoses in the dementia disease initiation and Amsterdam dementia cohorts

	Dementia Disease Initiation Aβ/Tau groups (*n*)				Post-hoc comparisons (*p*)
	CN Aβ-/Tau- 154	Aβ+/Tau- 45	Aβ+/Tau + 147	*F* / *χ*^*2*^ / *η*^*2*^ *(p)*	Aβ+/Tau- vs. Aβ+/Tau+
**Age** Mean (SD)	58.91 (8.62)	65.96*** (7.59)	67.79*** (7.64)	*F* = 47.51, *η*^*2*^ = 0.22 (**< 0.001**)	n.s^a^
**Female** n (%)	85 (55%)	33 (73%)	74 (50%)	*χ*^*2*^ = 7.38, **(< 0.05**)	^b^
**APOE-**ε4 carrier status (%) [n]	49 (32%) [152]	31 (70%) [44]	113 (78%) [145]	*χ*^*2*^ = 67.03, (**< 0.001**)	^b^
**Diagnoses**				*χ*^*2*^ = 160.42, (**< 0.001**)	^b^
**Recruited as controls** (n %)	50 (32%)	5 (11%)	16 (11%)		^b^
**SCD** (n %)	104 (68%)	17 (38%)	30 (20%)		^b^
**MCI** (n %)	0 (0%)	23 (51%)	101 (69%)		^b^

	Amsterdam Dementia Cohort Aβ/Tau groups (n)				Post-hoc comparisons (p)
	CN Aβ-/Tau- 187	Aβ+/Tau- 95	Aβ+/Tau + 115	*F* / *χ*^*2*^ / *η*^*2*^ *(p)*	Aβ+/Tau- vs. Aβ+/Tau+
**Age** Mean (SD)	64.01 (11.83)	66.86 (9.55)*	67.65 (7.96)**	*F* = 5.18, *η*^*2*^ = 0.03 **(0.006)**	n.s^a^
**Female** n (%)	76 (41%)	40 (42%)	53 (46%)	*χ*^*2*^ = 0.87, **(n.s)**	^b^
**APOE-**ε4 carrier status (%) [n]	50 (30%) [166]	60 (69%) [87]	75 (69%) [108]	*χ*^*2*^ = 54.9, **(< 0.001)**	^b^
**Diagnoses**					
**SCD** (n %)	187 (100%)	60 (63%)	47 (41%)	*χ*^*2*^ = 137.3, **(< 0.001)**	^b^
**MCI** (n %)	0 (0%)	35 (37%)	68 (59%)		

Abbreviations: Aβ +/-, positive or negative CSF marker for Aß plaques (DDI: Aß42/40 ratio; ADC Aß1–42); Tau +/-, positive or negative marker for either CSF p-tau181 and/or total-tau; SD, standard deviation; n, number of cases; %, percentage; F, F statistic; *χ*^*2*^, chi square statistic; *η*^*2*^, eta-squared; vs., versus; SCD, Subjective Cognitive Decline; MCI, Mild Cognitive Impairment; ^a^, ANOVA post-hoc (FDR adjustment); ^b^, no post-hoc comparisons performed; *, < 0.05,**, < 0.01, ***<0.001 (compared to the CN Aβ-/Tau- group)

### Measurement of CSF synaptic proteins

For DDI, synaptic proteins were measured with an in-house mass spectrometry assay. For comprehensive sample preparation details, please refer to the previously published work [33]. In summary, 25 µL of internal standard (heavy standard peptides conc see supplementary table 1A, JPT Peptide Technologies, Berlin, Germany; SpikeTides L) were added to 100 µL of CSF samples, followed by reduction, alkylation, digestion and desalting. The quantification of a panel of 16 synaptic and lysosomal proteins, was carried out using multiple reaction monitoring (MRM) with a micro-high-performance liquid chromatography tandem mass spectrometry system (Agilent Technologies, 6495 Triple Quadrupole LC/MS system) equipped with a Hypersil Gold reversed-phase column (Thermo Fisher Scientific, 100 × 2.1 mm, 1.9 μm particle size). Additional information on the MRM settings used can be found in supplementary table 1A. To monitor the performance of the assay, two different quality control (QC) samples consisting of CSF pools were periodically injected, one utilized for plate correction and the other for evaluation of the final analytical performance. The analysis exhibited high precision for most proteins within and between runs with a CV below 16.25% (supplementary table 1B). Mass spectrometric data analysis was conducted using Skyline 20.1 software (MacCoss Lab Software) where each peak was adjusted and visually inspected. Two hundred six data points were excluded during the study (0.1% missing data points) due to different reasons such as signal below limit of quantification, technical issues, and signal interference. GAP-43 was measured using an in-house ELISA at the University of Gothenburg as previously described [58]. 

In ADC, synaptic protein measurements were selected from a large-scale CSF proteomics analysis. Proteomics was performed using TMT-MS with 15-plexing, using a high pH reverse phase HPLC for peptide prefractionation. Reference channels were used to normalize peptide relative abundances between TMT experiments, according to standard procedures [59]. Details regarding CSF sample preparation and mass spectrometry analysis are previously published [60]. 

### Magnetic resonance imaging acquisition and post-processing

Magnetic resonance imaging **(**MRI) data from six DDI sites, acquired across four 1.5T and three 3.0T MRI systems, were included in the study. A subsample of 180 of 346 cases (52%) had available MRI in conjunction with CSF synapse markers (CN Aβ-/Tau-, *n* = 82; Aβ+/Tau-, *n* = 21 [CN = 10, MCI = 11]; Aβ+/Tau+, *n* = 77 [CN = 19, MCI = 58]). All MRI analyses were based on volumetric T1-weighted scans and the Automatic Segmentation of Hippocampal Subfields (ASHS) pipeline for total MTL volumes were used to quantify the entorhinal cortices (volumes in ASHS), anterior and posterior hippocampal volumes. ASHS provides fully automated segmentation, and the software is free, open-source and available online (https://www.nitrc.org/projects/ashs/). For the purposes of the present study, the included volumes were averaged across the hemispheres. All images were manually inspected by trained physicians (J. Jarholm, P. Selnes) for quality and correct segmentation after postprocessing. Please see Jarholm et al. [5], for further details on the included DDI MRI sites and scanners used in the present study.

### Verbal episodic memory assessments

Verbal episodic memory impairment is acknowledged as one of the earliest clinical features of AD [61]. In this study we focus on the earliest clinical stages of AD, including links to medial temporal lobe atrophy. Thus, only delayed verbal memory recall was included in our analyses. In DDI, the Norwegian version of the CERAD word list delayed memory recall subtest was used for pertinent associations with the synapse biomarkers. In ADC, the delayed recall subtest from the Dutch version of the Rey auditory verbal learning test (RAVLT) was used [62]. 

### Statistical analysis

All analyses were performed in RStudio (R version 4.3.2) [63]. Between-group (Aβ/Tau status) comparisons of age (dependent variable) was performed using a linear regression model. Post-hoc comparisons were adjusted using the False Discovery Rate (FDR) method. Group differences in nominal variables (sex and diagnostic group (CN vs MCI)) were assessed using chi-squared tests. Between-group comparisons of CSF synapse markers (dependent variables) were conducted using linear regression models in both the DDI and ADC cohorts separately. In these models, the dependent variables (CSF synapse markers) were log-transformed prior to analyses. The independent variables included Aβ/Tau group status (Aβ+/Tau- and Aβ+/Tau + groups compared to the reference CN Aβ-/Tau- group), with age and sex included as covariates. For each synapse marker, three linear regression models were fitted: (1) all Aβ + groups, (2) CN Aβ + groups and (3) MCI Aβ + groups as compared to CN Aβ-/Tau- group. Associations between synapse markers and episodic memory performance were tested using linear regression models, stratified by Aβ/Tau group. In these models, cognitive test scores (CERAD delayed recall in DDI; RAVLT delayed recall in ADC) served as the dependent variables, and each CSF synapse marker (log-transformed) was included as the main predictor, with age, sex, and education as covariates. FDR adjustments were applied within each model (17 tests). Pooled effect sizes across cohorts for both between-group differences in synapse marker concentrations and associations with memory performance were estimated using random-effects meta-analyses (metacont(), R package “meta”) [64], with heterogeneity assessed via I² and between-cohort variance via τ². Within DDI, baseline associations between each synapse marker (independent variable) and MRI regions of interest (dependent variables, entorhinal cortex thickness, posterior and anterior hippocampal volumes) were also assessed in a similar manner including FDR adjustments as outlined above (17 tests per model). To harmonize data from different scanners, we used the neuroCombat R package using an empirical Bayesian approach [65]. Harmonizations were applied for each model and synapse marker, including age, sex and intracranial volumes (ICVs) as covariates. For all linear regression models outlined above, both dependent and independent variables were z-standardized prior to analyses. Thus, standardized Z (between-group comparisons) or *β* coefficients (continuous associations) are reported.

Following the cross-sectional analyses, we employed a funneling procedure to select proteins most significantly associated with Aβ/Tau pathology and concurrent memory impairment observed across our two independent cohorts. This procedure focused on identifying proteins linked to pathological biomarkers and cognitive decline in cases compared to controls. These proteins were further assessed for associations to future memory decline in both DDI and ADC cohorts, to Aβ/Tau biological progression in the DDI cohort (no adjustment for multiple comparisons) and for associations to genetic pathways with established links to AD. Longitudinal associations between baseline CSF synapse markers (independent variables) with memory decline (dependent variable) were assessed using linear mixed models with random slopes for time for all cases in both the complete sample and split by Aβ/Tau groups. Age, sex, education and baseline cognitive status (CN/MCI) were included as covariates. Differences in baseline synapse marker concentrations (dependent variables) for the Aβ/Tau progressors were compared to stable CN and Aβ-/Tau- controls using linear regression. The models included a covariate for time since baseline. Plots were created using the ggeffects, ggpubr and ggplot2 R packages [63, 66, 67]. 

### Pathway analysis

The genes coding for the funneled proteins were found using GeneCards (https://www.genecards.org/). Using Python 3.11.2 and the packages requests 2.28.1 and json, The gene names were used in a Python script utilizing Enrichr’s REST API to query the BioPlanet database [68], and the resulting pathways were retrieved. Pathways with an adjusted p-value > 0.05 were discarded. The full Python-script can be found at github.com/ArcticPeAr/SynapticProteinsScripts.

## Results

### Between-group characteristics

We included 346 individuals from DDI (*n* = 154 CN Aβ-/Tau-, *n* = 45 Aβ+/Tau-, *n* = 147 Aβ+/Tau+) and 397 from ADC (*n* = 187 CN Aβ-/Tau-, *n* = 95 Aβ+/Tau-, *n* = 115 Aβ+/Tau+). In both cohorts, Aβ + groups were older compared with CN Aβ-/Tau- individuals (DDI: *p* < 0.001, ADC: *p* = 0.006) and more often carried an apolipoprotein E ε4 allele (both cohorts: *p* < 0.001). Details can be found in Table 1.

### Synapse marker concentrations in Aβ + subgroups

In the DDI cohort, 11 out of 17 proteins exhibited significantly lower concentrations (*p* < 0.05 to *p* < 0.001) in the Aβ+/Tau- group than in the CN Aβ-/Tau- reference group. However, an increase was noted for 14-3-3ζ/δ (*p* < 0.05). In the Aβ+/Tau + group, nearly all proteins (16 out of 17) showed higher concentrations compared to the CN Aβ-/Tau- reference group.

Similarly, in the ADC cohort 9 out of 17 proteins demonstrated lower concentrations in the Aβ+/Tau- group than in the CN Aβ-/Tau- reference group. Elevated concentrations were found for two proteins: 14-3-3ζ/δ and 14-3-3ε. Like in DDI, the Aβ+/Tau + group in ADC showed higher concentrations in 14 out of 17 proteins. Among the proteins with the lowest concentrations in both cohorts were NPTX2, NPTXR, VGF, and secretogranin-2. Conversely, the biomarkers with the most prominent increase included 14-3-3ζ/δ, 14-3-3ε, GAP-43, neurogranin, and GDI-1 (all *p* < 0.001).

**Fig. 1:**
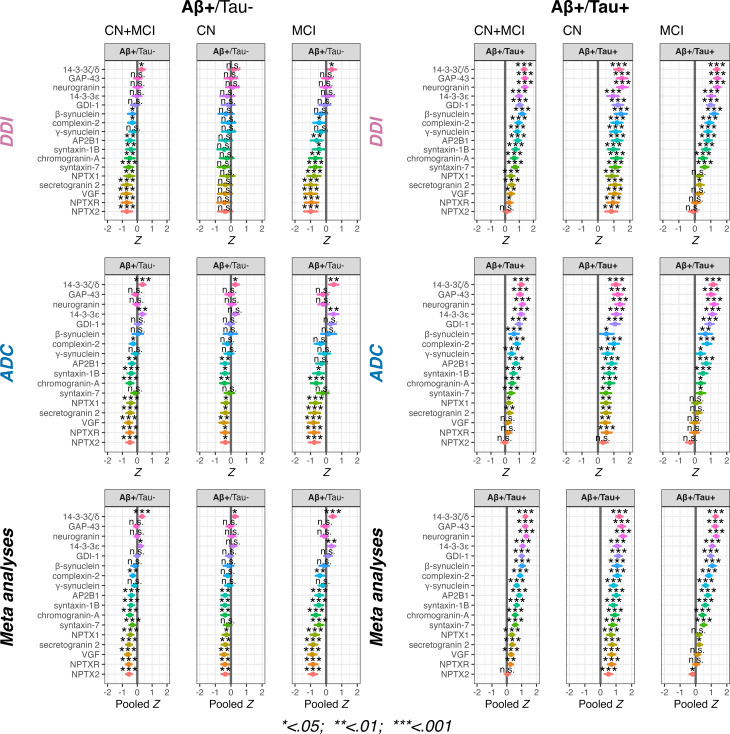
Between-group comparisons of synapse markers compared to controls. Between-group comparisons of synaptic marker concentrations relative to controls. Comparisons were made within Aβ+/Tau and Aβ+/Tau + groups, separately for cognitively normal (CN), mild cognitive impairment (MCI), and combined (CN + MCI) cases. The top row shows results from the Dementia Disease Initiation (DDI) cohort, the middle row from the Amsterdam Dementia Cohort (ADC), and the bottom row presents pooled estimates from meta-analyses across cohorts.The grey vertical bar in each plot is the reference group (CN Aβ-/Tau- controls). The top row are results from the Dementia Disease Initiation (DDI) cohort. The bottom row are results from the Amsterdam Dementia Cohort (ADC). *P*-values are listed as *, **, *** and n.s. corresponding to *p* < 0.05, < 0.01, < 0.001 and non-significant respectively. Horizontal bars show the 95% confidence intervals for each marker. All models included age and sex as covariates. Multiple comparisons were adjusted with False Discovery Rate

Pooled estimates through meta-analyses indicated 10 of 17 markers with significantly lower concentrations across cohorts in the Aβ+/Tau- group, and elevated concentrations for both 14-3-3ζ/δ and 14-3-3ε. For the Aβ+/Tau + group, all markers apart from NPTX2 showed significantly elevated concentrations. These meta-analyses showed low heterogeneity (all I² ≈ 0) and between-cohort variance (all τ²<0.02). Detailed results can be found in supplementary Tables 2 and Fig. 1.

### Synapse marker concentrations in Aβ+/Tau- CN and MCI subgroups

No significant differences were observed between CN Aβ+/Tau- and CN Aβ-/Tau- in the DDI cohort. However, a trend towards numerically lower concentrations was observed for several markers, as well as a numerical shift towards higher concentrations for 14-3-3ζ/δ. In ADC, we observed significantly lower concentrations in the CN Aβ+/Tau- group for 8 of 17 markers (AP2B1, syntaxin-1B, chromogranin-A, NPTX1, NPTX2, NPTXR, VGF and secretogranin-2), and also significantly increased 14-3-3ζ/δ (*p* < 0.05). In MCI Aβ+/Tau-, lower concentrations were observed for many of the same markers in DDI (10 of 17 markers) and ADC cohorts (7 of 17 markers) as compared to CN Aβ-/Tau-. Among the lowest biomarker concentrations in both groups were NPTX1, NPTX2, NPTXR, VGF and secretogranin-2 (all *p* < 0.001 in both cohorts). In both DDI and ADC higher concentrations were also observed for 14-3-3ζ/δ (*p* < 0.05; *p* < 0.01). Please see Fig. 1 and supplementary Table 2 for details.

Pooled estimates through meta-analyses indicated 8 of 17 markers with significantly lower concentrations across cohorts in the CN Aβ+/Tau- subgroup, and elevated concentrations for 14-3-3ζ/δ. In the MCI Aβ+/Tau- subgroup 10 of 17 markers were significantly lower, whereas both 14-3-3ζ/δ and 14-3-3ε were elevated. Although heterogeneity between cohorts was low (all I² ≈ 0), elevated between-cohort variance was observed for 2 of 17 markers in the CN Aβ+/Tau − subgroup (τ² = 0.023–0.086) and 6 of 17 markers in the MCI Aβ+/Tau- subgroup (τ² = 0.011–0.079). This likely reflects reduced precision due to smaller sample sizes in these subgroups. Detailed results can be found in supplementary Tables 2 and Fig. 1.

### Synapse marker concentrations in Aβ+/Tau + CN and MCI subgroups

Compared to the CN Aβ-/Tau- group, all 17 markers were significantly higher in CN Aβ+/Tau + in DDI and all but one in ADC. In contrast, MCI Aβ+/Tau + cases in both DDI and ADC showed seemingly similar concentrations for NPTX1, NPTX2, NPTXR and VGF as CN Aβ-/Tau-, but the same pattern of higher concentrations for the other biomarkers. Please see Fig. 1 and supplementary Table 2 for details.

Pooled estimates from meta-analyses indicated significantly elevated concentrations of all synaptic markers in the CN Aβ+/Tau + group. In the MCI Aβ+/Tau + group, all markers were significantly elevated except for VGF, NPTXR, NPTX1, and NPTX2. Although heterogeneity between cohorts was low (all I² ≈ 0), elevated between-cohort variance was observed for 11 of 17 markers in the CN Aβ+/Tau + subgroup (τ² = 0.011–0.109), and for 6 of 11 markers in the MCI Aβ+/Tau + subgroup (τ² = 0.006–0.090). This likely reflects reduced precision due to smaller subgroup sizes across cohorts. Detailed results can be found in supplementary Table 2 and Fig. 1.

### Associations between synapse markers and verbal episodic memory performance

**Fig. 2:**
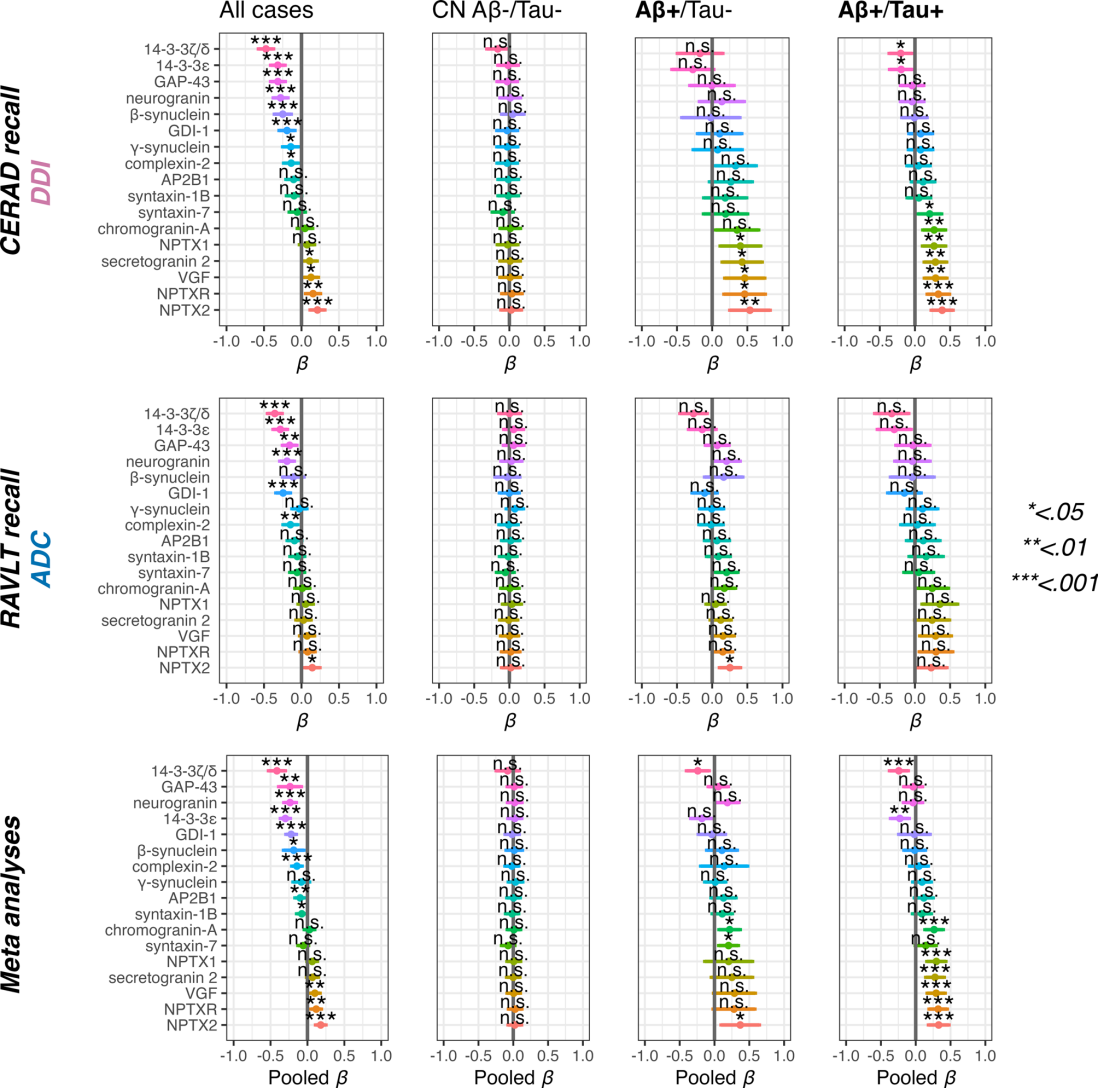
Associations between synapse markers and cross-sectional verbal memory performance. Associations between synaptic markers and cross-sectional verbal memory performance. Results are shown separately for the Dementia Disease Initiation (DDI) cohort (top row; CERAD verbal memory recall) and the Amsterdam Dementia Cohort (ADC; middle row; Rey Auditory Verbal Learning Test, RAVLT), with pooled meta-analytic estimates presented in the bottom row. The grey vertical bar in each plot is standardized beta (*β*) = 0. *P*-values are listed as *, **, *** and n.s. corresponding to *p* < 0.05, < 0.01, < 0.001 and non-significant respectively. Horizontal bars show the 95% confidence intervals for each marker. All models included age, sex and education as covariates. Multiple comparisons were adjusted with False Discovery Rate

In both DDI and ADC, we found that higher 14-3-3ζ/δ, 14-3-3ε, GAP-43, neurogranin, GDI-1 and complexin-2 (independent variables) all related to reduced memory performance (dependent variable) in the complete sample (all Aβ/Tau groups), as did β-synuclein and γ-synuclein in DDI. We also found that lower NPTX2 was related to reduced memory performance in both DDI (*p* < 0.001) and ADC (*p* < 0.01), as well as VGF, secretogranin-2 and NPTXR in DDI (between *p* < 0.01 and *p* < 0.05). In the Aβ+/Tau- group, lower NPTX2 was associated with worse memory performance in both DDI (*p* < 0.01) and ADC (*p* < 0.001) cohorts. In addition, lower NPTX1, NPTXR, VGF and secretogranin-2 were associated with worse memory in the DDI Aβ+/Tau- group (*p* < 0.05). Within the Aβ+/Tau + group, lower syntaxin-7, chromogranin-A, NPTXR, NPTX1, NPTX2, secretogranin-2, VGF and higher 14-3-3ζ/δ and 14-3-3ε were associated with worse memory, but only reaching statistical significance in the DDI cohort (please see Fig. 2 and supplementary Table 3).

Pooled estimates from meta-analyses showed that higher levels of 14-3-3ζ/δ, 14-3-3ε, GAP-43, neurogranin, GDI-1, complexin-2, β-synuclein, AP2B1, and syntaxin-1B, as well as lower levels of VGF, NPTXR, and NPTX2, were associated with reduced memory performance across all Aβ/Tau groups. In the Aβ+/Tau − group, higher 14-3-3ζ/δ and lower chromogranin-A, syntaxin-7, and NPTX2 were associated with reduced memory performance. In the Aβ+/Tau + group, higher 14-3-3ζ/δ and neurogranin, and lower chromogranin-A, secretogranin 2, VGF, NPTX1, NPTXR, and NPTX2, were associated with reduced memory performance. Heterogeneity between cohorts was low (all I² ≈ 0); however, 6 of 11 markers in the Aβ+/Tau − group showed elevated between-cohort variance (τ² = 0.028–0.049), likely reflecting reduced precision due to smaller sample sizes in this group compared to the Aβ+/Tau + subgroup. Please see Fig. 2 and supplementary Table 3.

### Associations between the synapse markers and MRI regions of interest

**Fig. 3:**
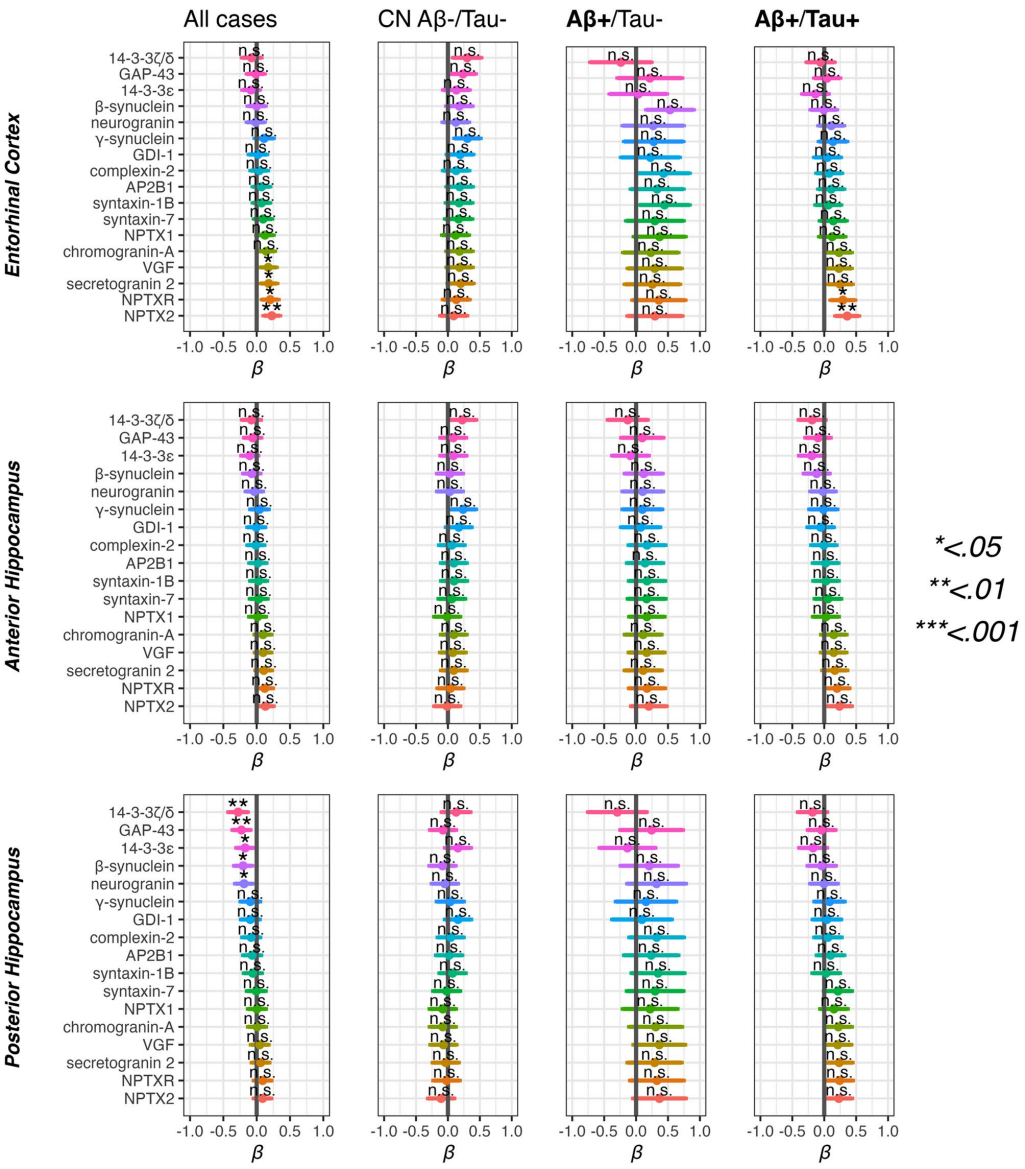
Associations between synapse markers and MRI regions of interest in Aβ/Tau groups. Associations between MRI medial temporal lobe regions of interest and synapse markers in the Aβ+/Tau- and Aβ+/Tau + and CN Aβ-/Tau- control groups. The grey vertical bar in each plot is standardized beta (*β*) = 0. All results are from the Dementia Disease Initiation (DDI) cohort. The top row: entorhinal cortex, middle row: anterior hippocampus, bottom row: posterior hippocampus. *P*-values are listed as *, **, *** and n.s. corresponding to *p* < 0.05, < 0.01, < 0.001 and non-significant respectively. All models included age, sex and intracranial volume as covariates. Multiple comparisons were adjusted with False Discovery Rate

We observed an association between increased 14-3-3ζ/δ (*p* < 0.01), 14-3-3ε (*p* < 0.01), GAP-43 (*p* < 0.05), β-synuclein (*p* < 0.05), and neurogranin (*p* < 0.05) and reduced posterior hippocampus volumes in the complete sample (all Aβ/Tau groups), but not within any of the Aβ/Tau subgroups. No significant associations between any of the synapse markers and anterior hippocampus volumes were found. Lower NPTX2 (*p* < 0.01), NPTXR (*p* < 0.05), secretogranin-2 (*p* < 0.05) and VGF (*p* < 0.05) were associated with thinner entorhinal cortices in the complete sample (all Aβ/Tau groups). An association between lower NPTX2 (*p* < 0.01) and NPTXR (*p* < 0.05) and thinner entorhinal cortices was also found in the Aβ+/Tau + group, but not in the CN Aβ-/Tau- or Aβ+/Tau- groups. Please see Fig. 3 and supplementary Table 4 for details.

The MRI subsample consisted of CN Aβ-/Tau- (*n* = 82), CN Aβ-/Tau- (*n* = 8), MCI Aβ+/Tau- (*n* = 13), CN Aβ+/Tau- (*n* = 15), and MCI Aβ+/Tau+ (*n* = 62). Because subgroup sizes were too small for stratified analyses of associations with synaptic markers, we instead examined between-group differences our MRI regions of interest. Relative to CN Aβ-/Tau- controls, MCI—irrespective of tau status—showed a pattern of lower posterior hippocampal volumes and thinner entorhinal cortex (Tau-, *p* < 0.05; Tau+, *p* < 0.001). In the anterior hippocampus, only the MCI Aβ+/Tau + group demonstrated significant volume loss (*p* < 0.001). These findings corresponded to worse memory performance in both MCI groups as compared to CN Aβ-/Tau- controls. Please see supplementary Table 5 for details.

### Funneling procedure to select pertinent synapse markers for association with disease progression

We performed a funneling procedure to select the proteins most significantly related to Aβ and Tau pathology as compared to CN Aβ-/Tau- and with links to memory impairment at baseline. Only results observed in both the DDI and ADC cohorts (Table 2) supported our selection of proteins. Following this procedure 14-3-3ζ/δ, 14-3-3ε, GAP-43, neurogranin, GDI-1 (negatively related to memory recall), and complexin-2 and NPTX2 (positively related to memory recall) were selected as candidates for association with disease progression.

**Table 2:** Selection of markers with change in either **Aβ+/**Tau- and/or **Aβ+/Tau +** as compared to Aβ-/Tau- and associations to verbal memory recall replicated in both the DDI and ADC cohorts

Synapse marker	Aβ+/Tau-			Aβ+/Tau+			Associations to memory recall				Selected for analyses of clinical and biological progression
	All	CN	MCI	All	CN	MCI	All	Aβ-/Tau-	Aβ+ /Tau-	Aβ+ /Tau+	
14-3-3 ζ/δ	↑	-	↑	↑	↑	↑	↓↓	-	-	-	✓
14-3-3-ε	-	-	-	↑	↑	↑	↓↓	-	-	-	✓
GAP43	-	-	-	↑	↑	↑	↓↓	-	-	-	✓
Neurogranin	-	-	-	↑	↑	↑	↓↓	-	-	-	✓
GDI-1	-	-	-	↑	↑	↑	↓↓	-	-	-	✓
Complexin-2	↓	-	-	↑	↑	↑	↓↓	-	-	-	✓
NPTX2	↓	-	↓	-	-	-	↑↑	-	↑↑	-	✓
β-synuclein	-	-	-	↑	↑	↑	-	-	-	-	
γ-synuclein	-	-	-	↑	↑	↑	-	-	-	-	
AP2B1	↓	-	-	↑	↑	↑	-	-	-	-	
Syntaxin-1B	↓	-	↓	↑	↑	↑	-	-	-	-	
Syntaxin-7	-	-	-	↑	↑	↑	-	-	-	-	
Chromogranin-A	↓	-	↓	↑	↑	↑	-	-	-	-	
NPTX1	↓	-	↓	↑	↑	-	-	-	-	-	
Secretogranin 2	↓	-	↓	↑	↑	-	-	-	-	-	
VGF	↓	-	↓	-	↑	-	-	-	-	-	
NPTXR	↓	-	↓	-	↑	-	-	-	-	-	

Abbreviations: Aβ +/-, positive or negative CSF marker for Aß plaques; Tau +/-, positive or negative marker for either CSF p-tau181 and/or total-tau; ↑, Elevated compared to Aβ-/Tau-; ↓, Elevated compared to Aβ-/Tau-; ↑↑, negative associations with memory recall; ↓↓, positive associations with memory recall

### Elevated 14-3-3ζ/δ, and lower NPTX2 in Aβ + MCI compared to Aβ + CN groups regardless of tau pathology

In our main between-group comparisons, we observed that several synaptic biomarkers may be reduced in MCI cases with Aβ+/Tau- and Aβ+/Tau + pathology compared to their CN counterparts (see Fig. 1). To further explore these patterns, we formally tested whether selected synaptic markers differed significantly between CN and MCI within Aβ + subgroups, based on the funneling procedure. Meta-analyses were also conducted to assess pooled effect sizes across cohorts. No post-hoc correction for multiple comparisons was applied.

**Fig. 4:**
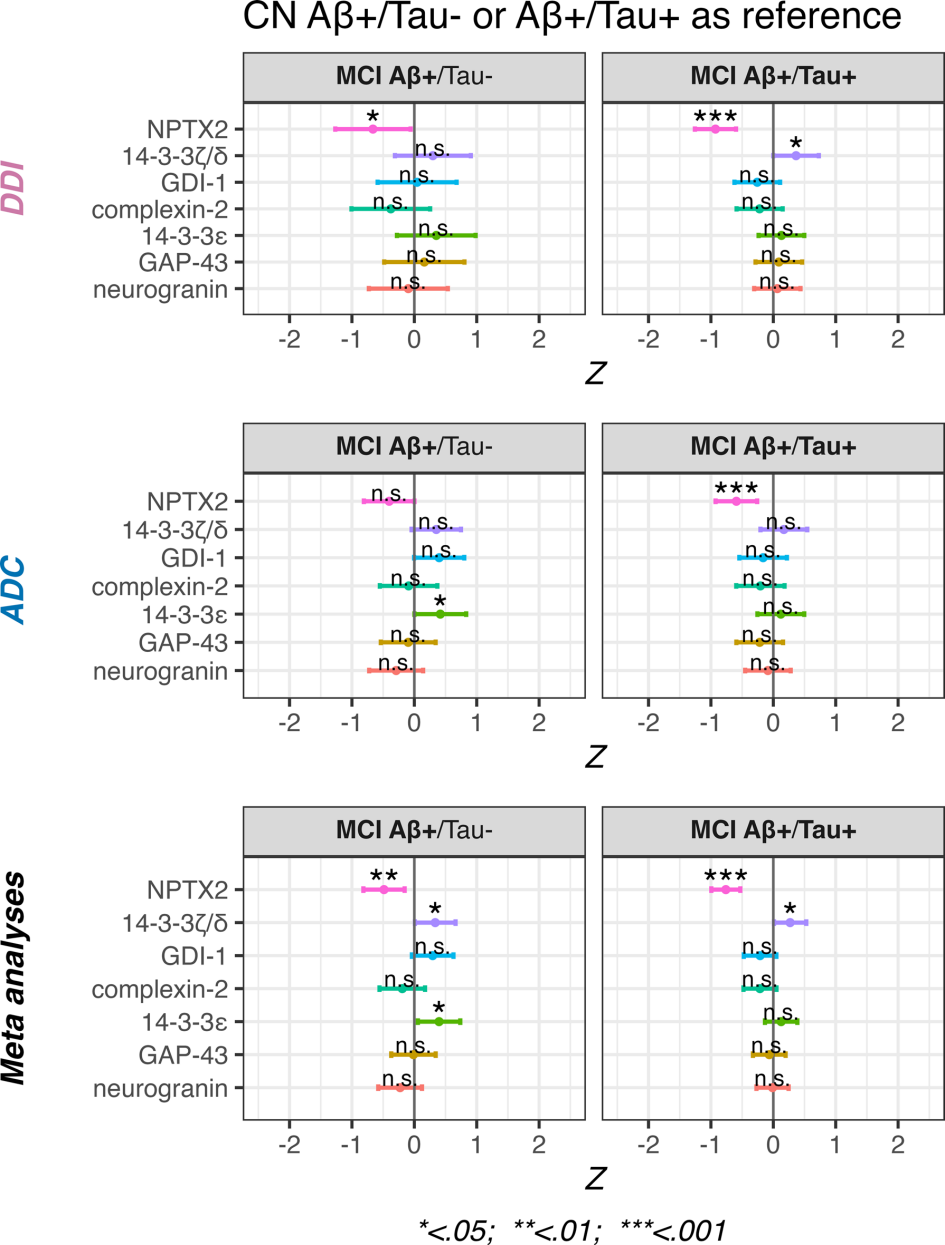
Within-pathology group comparisons of synaptic marker concentrations between cognitively normal and mild cognitive impairment cases. Analyses are restricted to synaptic proteins selected through our funneling approach. Comparisons were conducted separately within the Aβ+/Tau − and Aβ+/Tau + groups. The top row shows results from the Dementia Disease Initiation (DDI) cohort, the middle row from the Amsterdam Dementia Cohort (ADC), and the bottom row presents pooled effect estimates from meta-analyses across cohorts. The vertical grey line represents the cognitively normal (CN) reference group within each pathology group. *P*-values are listed as *, **, *** and n.s. corresponding to *p* < 0.05, < 0.01, < 0.001 and non-significant respectively. Horizontal bars show the 95% confidence intervals for each marker. All models included age and sex as covariates. No adjustment for multiple comparisons were performed

Although NPTX2 concentrations were lower in MCI Aβ+/Tau- compared to CN Aβ+/Tau- across both cohorts, statistical significance was reached only in the DDI cohort (*p* < 0.05). Similarly, elevated 14-3-3ε concentrations were observed in both cohorts, but reached significance only in the ADC cohort (*p* < 0.05). For 14-3-3ζ/δ, neither cohort showed a significant difference individually, but the direction of effect was consistent. Pooled estimates from the meta-analyses indicated significantly lower NPTX2 concentrations (*p* < 0.01), and significantly elevated 14-3-3ζ/δ and 14-3-3ε levels (both *p* < 0.05) in the MCI Aβ+/Tau- group. Please see Fig. 4 and supplementary Table 6 for details.

NPTX2 concentrations were significantly lower in MCI Aβ+/Tau + as compared to CN Aβ+/Tau + in both DDI and ADC (both *p* < 0.001). In contrast, a significant elevation of 14-3-3ζ/δ was observed only in the DDI cohort (*p* < 0.05). However, pooled estimates from the meta-analyses confirmed significantly lower NPTX2 concentrations (*p* < 0.001) and also revealed a significant elevation of 14-3-3ζ/δ (*p* < 0.05) in the MCI Aβ+/Tau + group. Please see Fig. 4 and supplementary Table 6 for details.

### Correlations between core AD biomarkers and the synaptic markers

We conducted a supplementary analysis of bivariate Spearman’s roh correlations between core CSF AD markers and the synaptic markers prioritized by the funneling procedure. Specifically, we correlated Aβ42/40 in DDI, Aβ1–42 in ADC, and p-tau181 and t-tau in both cohorts, estimating correlations in the full sample within each cohort. Overall, patterns of correlations were similar between cohorts. The Aβ markers were negatively correlated with all synaptic markers in both cohorts (all *p* < 0.001) except NPTX2 (p = n.s.). In contrast, all markers, including NPTX2 were positively correlated with the tau markers in both cohorts (all *p* < 0.001). Please see supplementary Fig. 1 for details.

### Longitudinal associations between selected synapse markers and future verbal memory decline

In both DDI and ADC cohorts, higher baseline levels of 14-3-3ζ/δ were consistently associated with memory decline (DDI: *β* = -0.045; ADC: *β* = -0.047, *p* < 0.001 for both). Similarly, increased levels of GDI-1 and GAP-43 were linked to decline in both cohorts, with GDI-1 showing *β* = -0.030 (DDI) and *β* = -0.034 (ADC), both *p* < 0.01; GAP-43 presented *β* = -0.041 (DDI, *p* < 0.001) and *β* = -0.026 (ADC, *p* < 0.05). Complexin-2 was significantly associated with memory decline in the DDI cohort (*β* = -0.027, *p* < 0.01), but not in ADC (*β* = -0.014, *p* = 0.175). Conversely, 14-3-3ε was a significant predictor in ADC (*β* = -0.040, *p* < 0.001) but not in DDI (*β* = -0.012, *p* = 0.252). Please see supplementary Table 7 for details.

Within the CN Aβ-/Tau- group, only the DDI cohort showed associations between higher concentrations of GAP-43, neurogranin, and NPTX2 with memory decline, with *β* values of -0.048 (*p* < 0.01 and *p* < 0.05 respectively) and − 0.038 (*p* < 0.05). In the Aβ+/Tau- group, 14-3-3ζ/δ and GDI-1 emerged as significant markers in ADC but not in DDI. For the Aβ+/Tau + group in DDI, 14-3-3ζ/δ alone was linked to memory decline (*β* = -0.040, *p* < 0.05).

**Fig. 5:**
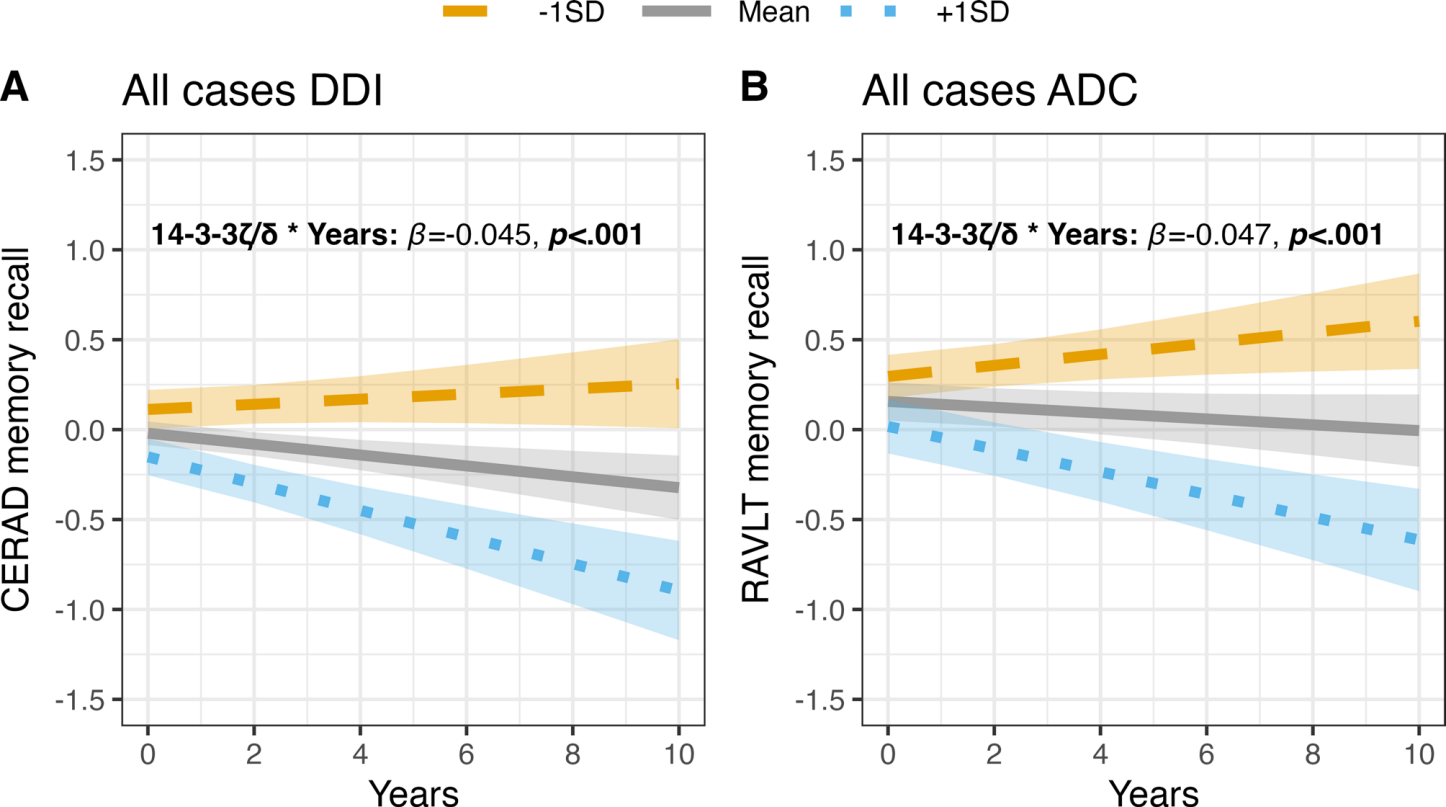
Associations between baseline CSF 14-3-3ζ/δ and future memory decline. Shows the longitudinal associations between baseline 14-3-3ζ/δ and future memory decline in (**A**) the Dementia Disease Initiation cohort (DDI, Consortium to Establish a Registry for Alzheimer’s Disease (CERAD) memory recall) and (**B**) the Amsterdam Dementia Cohort (ADC, Rey Auditory Verbal Learning Test (RAVLT) memory recall). Ribbons fitted to each regression line shows the 95% confidence interval for the estimates. The models included age at baseline, sex and education as covariates

Across both cohorts, 14-3-3ζ/δ proved to be the most robust and significant marker of future memory decline, particularly evident in the complete sample and the Aβ + groups (see Fig. 5 and supplementary Table 7).

### AD biological progression

**Fig. 6:**
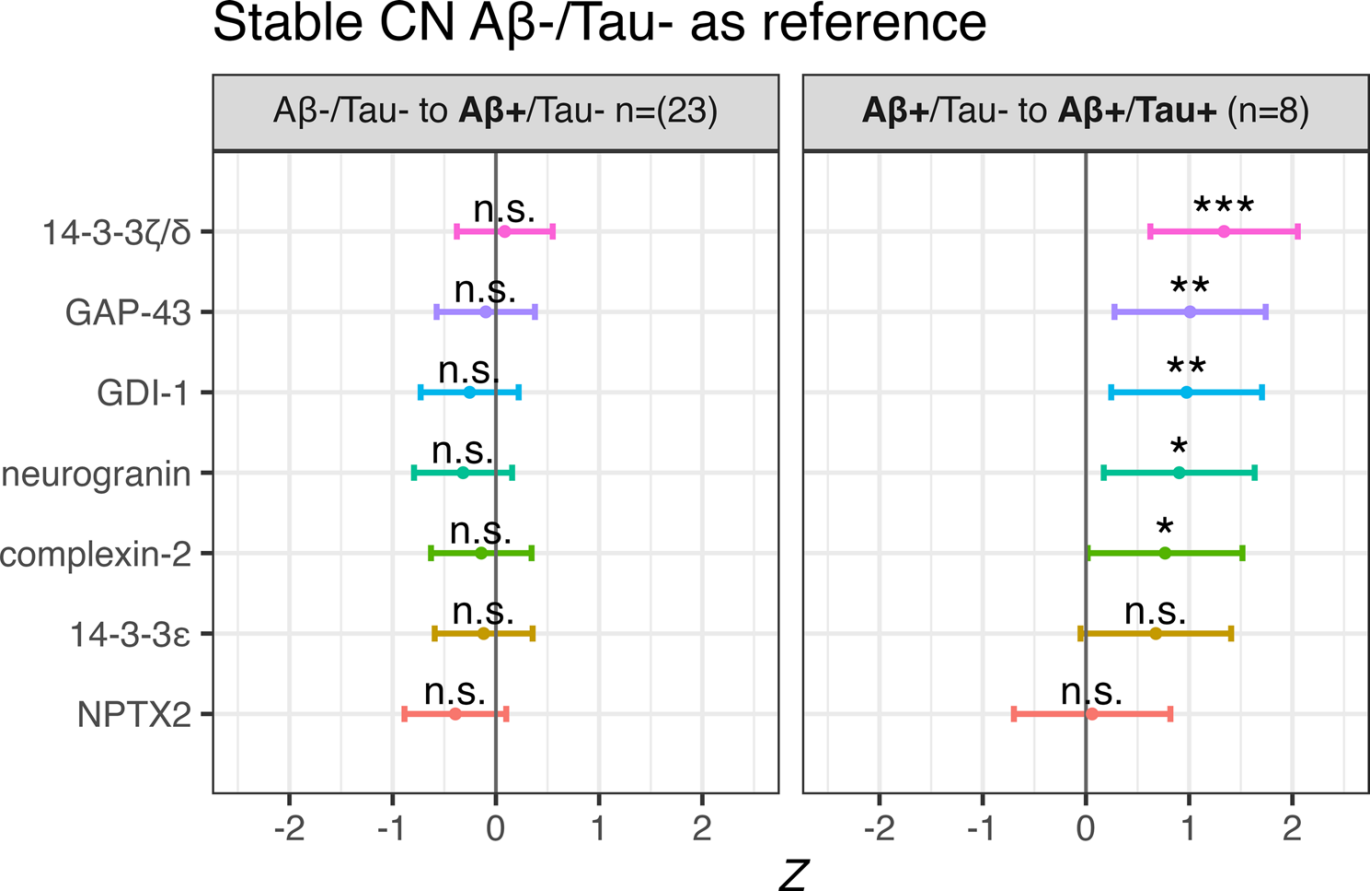
Comparison of baseline synapse markers with pathological Aβ and/or Tau progression. Comparison of baseline synapse marker concentrations in Dementia Disease Initiation (DDI) cases progressing from Aβ-/Tau- to Aβ+/Tau- (**A**) and from Aβ+/Tau- to Aβ+/Tau+ (**B**) as compared to stable cognitively normal (CN) and Aβ-/Tau- controls (vertical grey bar in each plot). *P*-values are listed as *,**,*** and n.s. corresponding to *p* < 0.05, < 0.01, < 0.001 and non-significant respectively. Horizontal bars show the 95% confidence intervals for each marker. All models included adjustment for individual years of observation. No adjustment for multiple comparisons were performed

In cases progressing from Aβ-/Tau- to Aβ+/Tau- over time (*n* = 23), no baseline differences in synapse marker concentrations were observed when compared to stable Aβ-/Tau- CN controls. In contrast, cases progressing from Aβ+/Tau- to Aβ+/Tau+ (*n* = 8) demonstrated significant baseline elevations in 14-3-3ζ/δ (β = 1.34, *p* < 0.001), followed by GAP-43 (*β* = 1.01, *p* < 0.01), GDI-1 (*β* = 0.98, *p* < 0.01), neurogranin (*β* = 0.90, *p* < 0.05) and complexin-2 (*β* = 0.77, *p* < 0.05) (see Fig. 6).

### Association to gene expression pathways

The genes coding for the proteins fulfilling the funneling criteria (Table 2) were *YWHAZ* (14-3-3ζ/δ) and *YWHAE* (14-3-3ε), *CPLX2* (complexin-2), *GAP43* (GAP-43), *NRGN* (neurogranin), *GDI1* (GDI-1), *NPTX2* (NPTX2). These were selected as targets for pathway enrichment analysis as described above. We found *YWHAZ* and *YWHAE* to be associated to pathways linked to AD. In particular to the “p38 alpha/beta MAPK downstream pathway” [69], to “Insulin regulation of blood glucose” [70]^45^, “IGF1 pathway” [71], ^“^PIK3C1/AKT pathway” [72], and “PI3K/PLC/TRK pathways” (all *p* < 0.001) [73]. *GDI-1* also showed an association to the “p38 alpha/beta MAPK downstream pathway”, *GAP43* to “N-cadherin signaling events” and *NRGN* to the “TSH regulation of gene expression pathway”, all the latter at *p* < 0.05 (please see Table 3 for a full list of significant associations). However, these results should be interpreted with caution given the low number of funneled proteins available for enrichment analyses. Particularly for *GAP43*, *NRGN* and *GDI1* which were only associated with single pathways.

**Table 3:** Bioplanet pathways found using the genes coding for the proteins selected for analyses

Pathway	Genes	Adjusted *p*-value	Odds ratio	Combined score	Proteins
p38 MK2 pathway	YWHAE, YWHAZ	6.45E-04	4.21E + 02	4.51E + 03	14-3-3 ζ/δ, 14-3-3-ε
Insulin regulation of blood glucose	YWHAE, YWHAZ	6.45E-04	3.19E + 02	3.26E + 03	14-3-3 ζ/δ, 14-3-3-ε
IGF1 pathway	YWHAE, YWHAZ	6.45E-04	2.96E + 02	2.98E + 03	14-3-3 ζ/δ, 14-3-3-ε
PIK3C1/AKT pathway	YWHAE, YWHAZ	6.45E-04	2.42E + 02	2.34E + 03	14-3-3 ζ/δ, 14-3-3-ε
PI3K/PLC/TRK pathway	YWHAE, YWHAZ	6.45E-04	2.35E + 02	2.26E + 03	14-3-3 ζ/δ, 14-3-3-ε
Alpha-6 beta-1 and alpha-6 beta-4 integrin signaling	YWHAE, YWHAZ	7.50E-04	1.81E + 02	1.66E + 03	14-3-3 ζ/δ, 14-3-3-ε
LKB1 signaling events	YWHAE, YWHAZ	7.50E-04	1.77E + 02	1.61E + 03	14-3-3 ζ/δ, 14-3-3-ε
FoxO family signaling	YWHAE, YWHAZ	7.50E-04	1.70E + 02	1.53E + 03	14-3-3 ζ/δ, 14-3-3-ε
Calcineurin-dependent NFAT signaling role in lymphocytes	YWHAE, YWHAZ	8.41E-04	1.50E + 02	1.32E + 03	14-3-3 ζ/δ, 14-3-3-ε
Nuclear beta-catenin signaling and target gene transcription regulation	YWHAE, YWHAZ	1.60E-03	1.02E + 02	8.19E + 02	14-3-3 ζ/δ, 14-3-3-ε
ERBB1 downstream pathway	YWHAE, YWHAZ	2.46E-03	7.65E + 01	5.71E + 02	14-3-3 ζ/δ, 14-3-3-ε
Oocyte meiosis	YWHAE, YWHAZ	2.46E-03	7.16E + 01	5.26E + 02	14-3-3 ζ/δ, 14-3-3-ε
mTOR signaling pathway	YWHAE, YWHAZ	2.46E-03	7.16E + 01	5.26E + 02	14-3-3 ζ/δ, 14-3-3-ε
Neurotrophin signaling pathway	YWHAE, YWHAZ	2.77E-03	6.41E + 01	4.56E + 02	14-3-3 ζ/δ, 14-3-3-ε
PDGFB signaling pathway	YWHAE, YWHAZ	2.77E-03	6.26E + 01	4.42E + 02	14-3-3 ζ/δ, 14-3-3-ε
Calcium regulation in the cardiac cell	YWHAE, YWHAZ	3.46E-03	5.40E + 01	3.66E + 02	14-3-3 ζ/δ, 14-3-3-ε
Myometrial relaxation and contraction pathways	YWHAE, YWHAZ	3.52E-03	5.19E + 01	3.48E + 02	14-3-3 ζ/δ, 14-3-3-ε
NADE-dependent death signaling	YWHAE	5.71E-03	6.66E + 02	4.11E + 03	14-3-3-ε
Glycoprotein 1b-IX-V activation signaling	YWHAZ	9.01E-03	3.70E + 02	2.09E + 03	14-3-3 ζ/δ
Rap1 signaling	YWHAZ	1.37E-02	2.22E + 02	1.15E + 03	14-3-3 ζ/δ
Destabilization of mRNA by KSRP	YWHAZ	1.38E-02	2.08E + 02	1.07E + 03	14-3-3 ζ/δ
Signaling by Hippo	YWHAE	1.55E-02	1.75E + 02	8.70E + 02	14-3-3-ε
Cell cycle	YWHAE, YWHAZ	2.12E-02	1.73E + 01	7.99E + 01	14-3-3 ζ/δ, 14-3-3-ε
N-cadherin signaling events	GAP43	2.40E-02	1.01E + 02	4.47E + 02	GAP43
GM-CSF-mediated signaling events	YWHAZ	2.40E-02	9.50E + 01	4.16E + 02	14-3-3 ζ/δ
p38 alpha/beta MAPK downstream pathway	GDI1	2.40E-02	8.99E + 01	3.89E + 02	GDI-1
Signaling events mediated by HDAC class II	YWHAE	2.40E-02	8.99E + 01	3.89E + 02	14-3-3-ε
Interleukin-3, interleukin-5, and GM-CSF signaling	YWHAZ	2.74E-02	7.56E + 01	3.14E + 02	14-3-3 ζ/δ
Pathogenic Escherichia coli infection	YWHAZ	3.34E-02	5.93E + 01	2.33E + 02	14-3-3 ζ/δ
Telomerase regulation	YWHAE	3.79E-02	5.03E + 01	1.89E + 02	14-3-3-ε
Prolactin activation of MAPK signaling	YWHAZ	4.10E-02	4.49E + 01	1.64E + 02	14-3-3 ζ/δ
mRNA stability regulation by proteins that bind AU-rich elements	YWHAZ	4.46E-02	3.90E + 01	1.37E + 02	14-3-3 ζ/δ
Mitotic G2-G2/M phases	YWHAE	4.46E-02	3.86E + 01	1.35E + 02	14-3-3-ε
TSH regulation of gene expression	NRGN	4.82E-02	3.45E + 01	1.17E + 02	Neurogranin

## Discussion

In this study, we examine a panel of 17 CSF synaptic biomarkers and give a comprehensive overview across predementia Aβ+/Tau+/- and clinical stages, investigate their association with cognitive functioning at baseline and over time as well as with neurodegeneration markers on volumetric MRI. Supported by meta-analyses of pooled effect sizes, our main findings are consistent across our two independent cohorts (DDI & ADC), showing distinct patterns of increased levels of several synapse markers in Aβ+/Tau + patients, in particular the 14-3-3ζ/δ isoforms. Interestingly, in Aβ+/Tau- patients, only the 14-3-3 proteins were increased, whereas levels for the NPTX2, NPTX1, NPTXR, VGF and secretogranin-2 group of proteins were consistently reduced across cohorts. However, the relative patterns of biomarker change in both Aβ+/Tau- and Aβ+/Tau + were similar. Notably, the relative increase in e.g. 14-3-3ζ/δ and reduction in e.g., NPTX2 was more pronounced in patients with MCI than in the preclinical CN cases. Overall higher levels of NPTX2, NPTXR, VGF and secretogranin 2 were linked to retained or higher ERC volumes in the Aβ + and Aβ+/Tau + group consistent with associations to stronger verbal memory recall. An inverse relationship was observed for the posterior hippocampus, where lower 14-3-3 levels corresponded to greater volumes. This finding was consistent with memory-related cross-sectional findings and with associations to longitudinal trends for verbal memory decline, with prominent effects of 14-3-3ζ/δ coming into play with more advanced pathology. The more prominently expressed changes seen in pathological Aβ+/Tau- MCI vs. CN-groups could reflect less versus higher degrees of resilience in these groups.

These findings are well in accord with a current understanding of the suggested roles these proteins play in the upholding and homeostasis of synaptic structures. However, the panel approach combined with multimodal analysis and longitudinal follow up adds essential new data and understanding on the involvement of subsets of synapse markers in AD predementia subgroups, narrowing down the critical mechanisms involved in synaptic pathology. Notably, no expression changes were detected in advance of Aβ pathology, consistent with induction of synaptic pathologies by core AD pathology.

NPTX2 binds complement C1q, deficiency increases complement activity associated with synapse loss [74] and could be related to both memory impairment and atrophy of hippocampal and sub-hippocampal cortices observed in this study. Consistent with a chaperone role in tau metabolism [29], highly significant links to main genetic pathways in AD may suggest mechanistic roles for 14-3-3 proteins in disease progression. Increased 14-3-3 expression may result from Aβ dysmetabolism but may also interact with Aβ clearance through effects on PI3K metabolism and direct associations with endolysosomal proteins [75]. Furthermore, the close associations of *YWHAZ* and *YWHAE* to the p38 MK2 pathways influencing synaptic plasticity and degeneration may point to a role for 14-3-3 proteins in disease progression [76]. The p38αMAPK pathway is downstream to glial activation and inflammation, but also induces innate immune activation and glutamatergic synaptic plasticity and is currently a target in AD drug development [77, 78]. However, also the links to the AKT and PI3K pathways involving GSK3, TSC2 and FOXO proteins impacting both tau phosphorylation and mTORC1 activation with effects on autophagy and cell-cycle progression are consistent with a role in progression towards more advanced AD pathology [72, 73]. 

Interestingly, the GDI-1 protein was also elevated at baseline in cases progressing from Aβ+/Tau- to Aβ+/Tau+, and we show significant associations to future memory decline. As *YWHAZ* and *YWHAE*, *GDI1* is connected to the “p38 alpha/beta MAPK downstream pathway”. GDI-1 inhibits Rab proteins that are involved in synaptic vesicle endocytosis/exocytosis and vesicle fusion of early and late endosomes [31]. Mutations in *GDI-1* are associated with intellectual disability, and genes for several Rab proteins have been linked to AD suggesting mechanistic effects of GDI-1 expression in disease progression [79]. 

Our results confirm earlier reports showing that increased GAP-43 is linked to increased tau-pathology, and we show significant links to future memory decline [80]. GAP-43 is linked to synaptic plasticity and (putatively) tau-spreading [80], and the pathway association analysis shows links to “N-cadherin signaling events”. Increased expression of C-terminal N-cadherin fragment 1, as found in AD brains, has been shown to accelerate Aβ-induced synapse impairment providing a putative mechanistic link between GAP-43 expression and disease progression [81]. 

Increased CSF neurogranin levels are well-documented in AD and show significant associations with tau pathology [82]. Here we confirm significant associations to increased tau-pathology and future memory decline (in DDI). Neurogranin is connected to the “TSH regulation of gene expression pathway”. Mechanistically, neurogranin is functionally coupled to glutamate NMDA signaling via Ca^2^+/calmodulin which is implicated in AD neurotoxicity [83]. 

Our findings may suggest the presence of two distinct processes along the predementia continuum of AD. Firstly, amyloid pathology appears primarily associated with a downregulation of specific groups of synaptic proteins. Secondly, tau pathology is linked to a broader upregulation of most synaptic proteins. We see that more extensive neurodegeneration linked to increased tau levels is accompanied by broadly increased release of synaptic proteins [17, 19]. Neurodegeneration will lead to deafferentation known to induce sprouting and microglial activation [84]. Thus, we propose that this second process would encompass a CNS plasticity response including microglial activation, putatively also linked to key pathways for synapse loss [20, 85–87]. These two processes are consistent with the recently proposed AD subtypes characterized by synaptic hypoplasticity (Aβ+/Tau-) or hyperplasticity (Aβ+/Tau+), both still being associated with significant clinical impairment and disease progression [60, 88]. Increased synaptic markers in Aβ+/Tau + cases may align with a neuronal hyperplasticity phenotype, whereas Aβ+/Tau- cases with reduced synaptic markers may constitute a hypoplasticity phenotype. Both the MCI Aβ+/Tau- and MCI Aβ+/Tau + groups show significant reductions in NPTX2, and elevation in 14-3-3ζ/δ compared to their CN counterparts. However, the concentration levels are far higher in Aβ+/Tau + cases and thus reductions in e.g. NPTX2 seen in Aβ+/Tau- MCI cases compared to Aβ-/Tau- are non-significantly altered in Aβ+/Tau + MCI cases. This may reflect more widespread neurodegeneration and synapse loss in Aβ+/Tau + cases [17]. These interpretations are supported by common biomarker links to cognitive impairment and associations to MRI atrophy patterns within Aβ+/Tau+/- groups and to AD-linked genetic pathways as discussed above. Higher levels of markers such as NPTX2 in cognitively normal Aβ+/Tau + individuals could reflect a more resilient phenotype with adaptive synaptic sprouting in a (premorbid) more elaborate synaptic network [89]. This interpretation is supported by previous research, which suggests that the neuronal hyperplasticity subtype may be associated with slower clinical progression [60]. 

We have previously shown that a 14-3-3ζ/δ/NPTX2 ratio best associates with future cognitive decline and brain atrophy [90]. Here we also show that both higher 14-3-3ζ/δ and lower NPTX2 jointly correspond to clinical impairment regardless of tau-pathology. Thus, the ratio is likely a sensitive marker of disease progression. Though both markers target synapse pathology, distinct mechanisms (as described above) are likely involved, putatively adding to the robustness of the ratio.

The primary strength of this study when comparing potential synaptic biomarkers across AD subgroups, lies in the use of multiplexed mass spectrometry and the availability of multiple candidate markers. This methodology enables the simultaneous measurement of multiple biomarkers allowing us to study a range of synaptic biomarkers with different functions and localizations. This approach not only facilitates comparison between biomarkers but also strengthens the credibility of results by validating common and specific pathological patterns among proteins. Using the longitudinal DDI cohort, we have been able to determine stages where biomarker changes and underlying mechanisms come into play. Furthermore, through the funneling procedure we have been able to single out significantly involved markers and pathways common to the development of synapse pathologies. Up to 9.67 (DDI) and 9.87 (ADC) years of longitudinal cognitive data were included for 79% (DDI) and 84% (ADC) of cases. Moreover, the findings were independently replicated across two relatively large cohorts – and supported by meta-analyses of pooled effect sizes (346 and 397 cases respectively) representing 1984 observations over time. The second (ADC) employed explorative mass spectrometry thus validating the initial results from DDI using an alternative quantification method.

As for limitations, it should be noted that only cross-sectional measurements of synaptic proteins were available at the time of analyses. However, we aim to address longitudinal analyses of synaptic markers in future work. While CSF p-tau181 and total-tau are highly correlated in AD, the use of a combined “Tau+” could have obscured minor differences in e.g. A+/T or (N) + profiles. Furthermore, because of a fairly strict funneling procedure for selection of proteins implicated in the AD process, only 7 genes were uploaded for enrichment analysis putting pathways with a high number of genes at a disadvantage [91]. With so few genes, we cannot robustly demonstrate that any pathway is enriched relative to an appropriate background; therefore, our pathway results are best viewed as annotations rather than definitive evidence of pathway involvement. Nevertheless, our results point to synapse pathology arising through involvement of a significant set of pathways already linked to AD. Further, the results suggest that synapse pathology arise downstream to amyloid pathology. Interestingly, proteins showing the strongest connections to pathological and cognitive progression connect to the same pathways. Future studies should focus on longitudinal analysis of synaptic markers to confirm the value of the biomarkers to track disease progression, delineate putative AD subtypes or contributions of neurodegeneration to biomarker concentrations. Furthermore, the innate immune system plays a pivotal role in synaptic homeostasis. Although the ADC mass-spectrometry data include measurements of innate immune markers, assessing their relationship with synaptic markers was beyond the scope of the current study. These analyses will be addressed in future work. Targeting of pertinent pathway-related mechanisms with downstream effects on synapses should also be further explored. We have initiated studies focusing on synaptic markers as downstream endpoints of AD pathologies.

## Conclusions

We demonstrate distinct CSF synapse marker profiles in AD that align with disease progression and genetic pathways. Our findings suggest that differential synaptic marker expression in Aβ+/Tau- and Aβ+/Tau + groups, linked to genetic pathways involved in both AD pathophysiology and synaptic plasticity, may reflect underlying processes of both synaptic loss and plasticity responses, putatively as a response to neurodegeneration.

If confirmed, these findings contribute to a more nuanced understanding of synaptic pathology in AD, offering new avenues for targeting synaptic dysfunction as potentially novel treatment targets in early disease stages. Future work is warranted to explore the possible mechanistic roles of these biomarkers and connected pathways, and validate their potential as markers of AD subtypes with differing synaptic plasticity profiles.

## Supplementary Information

Below is the link to the electronic supplementary material.

Supplementary Material 1

Supplementary Material 2: Supplementary table 1A and 1B. Description of data: Supplementary table 1A: LC-MS/MS settings for the analysis of the synaptic and lysosomal protein panel. Supplementary table 1B: LC-MS/MS concentrations of internal standard for each peptide as well as repeatability and intermediate precision for the study after plate correction.

Supplementary Material 3: Supplementary Table 2. Description of data: Detailed statistical results from between-group comparisons of synaptic marker concentrations in Aβ + groups relative to cognitively normal Aβ-/Tau- individuals, conducted separately in the Dementia Disease Initiation (DDI) and Amsterdam Dementia Cohort (ADC). The table also includes results from meta-analyses pooling effect sizes across cohorts.

Supplementary Material 4: Supplementary Table 3. Description of data: Detailed statistical results for associations between synaptic marker concentrations and baseline verbal memory impairment, analyzed separately in the Dementia Disease Initiation (DDI) and Amsterdam Dementia Cohort (ADC), with pooled estimates provided through meta-analyses across cohorts.

Supplementary Material 5: Supplementary Table 4. Description of data: Detailed statistical results for associations between synapse markers and Magnetic Resonance Imaging (MRI) Regions Of Interest (ROIs) in the Dementia Disease Initiation cohort.

Supplementary Material 6: Supplementary Table 5. Description of data: Detailed statistical results from within-pathology group comparisons of synaptic marker concentrations between cognitively normal and mild cognitive impairment cases, restricted to proteins identified through our funneling approach. Analyses were conducted separately within Aβ+/Tau- and Aβ+/Tau + groups in the Dementia Disease Initiation (DDI) and Amsterdam Dementia Cohorts (ADC), with pooled effect estimates provided through meta-analyses across cohorts.

Supplementary Material 7: Supplementary Table 6. Description of data: Detailed statistical results for associations between synapse markers and future verbal memory decline.

Supplementary Material 8

## Data Availability

Data from the DDI cohort are stored at Services for sensitive data (TSD) at the University of Oslo (UiO) and is publicly unavailable. However, anonymized data used in this study may be made available from the corresponding author upon reasonable request. ADC proteomic data is available through the ADDI (https://fair.addi.ad-datainitiative.org/#/data/datasets/five_csf_proteomic_subtypes_in_ad; 10.58085/HR6S-2991).

## Abbreviations

Aβ: Amyloid Beta
ADC: Amsterdam Dementia Cohort
ASHS: Automatic Segmentation of Hippocampal Subfields
CERAD: Consortium to Establish a Registry for Alzheimer’s Disease
CN: Cognitively Normal
COWAT: Controlled Oral Word Association Test
CSF: Cerebrospinal Fluid
DDI: Dementia Disease Initiation
ELISA: Enzyme-Linked Immunosorbent Assay
ERC: Entorhinal Cortex
FDR: False Discovery Rate
GAP-43: Growth Associated Protein 43
GDI-1: Rab GDP Dissociation Inhibitor Alpha
ICV: Intracranial Volume
MCI: Mild Cognitive Impairment
MRI: Magnetic Resonance Imaging
MTL: Medial Temporal Lobe
NPTX: Neuronal Pentraxin
NPTX1: Neuronal Pentraxin 1
NPTX2: Neuronal Pentraxin 2
NPTXR: Neuronal Pentraxin Receptor
PET: Positron Emission Tomography
P-tau: Phosphorylated Tau
RAVLT: Rey Auditory Verbal Learning Test
SCD: Subjective Cognitive Decline
SV2A: Synaptic Vesicle Glycoprotein 2 A
TMT-B: Trail Making Test, Part B
TMT-MS: Tandem Mass Tag Mass Spectrometry
T-tau: Total Tau
Tau: Tau Protein (Phosphorylated Tau and/or Total Tau)
VGF: Neurosecretory Protein VGF
VOSP: Visual Object and Space Perception Battery
