# Supplementary material for: Cerebrospinal fluid markers link to synaptic plasticity responses and Alzheimer’s disease genetic pathways
Source: Mol Neurodegener. 2025 Oct 13;20:107. doi: 10.1186/s13024-025-00899-w (PMC12519626; doi:10.1186/s13024-025-00899-w)
Supplement: Supplementary file 1 — Supplementary Material 1 [file 13024_2025_899_MOESM1_ESM.docx]

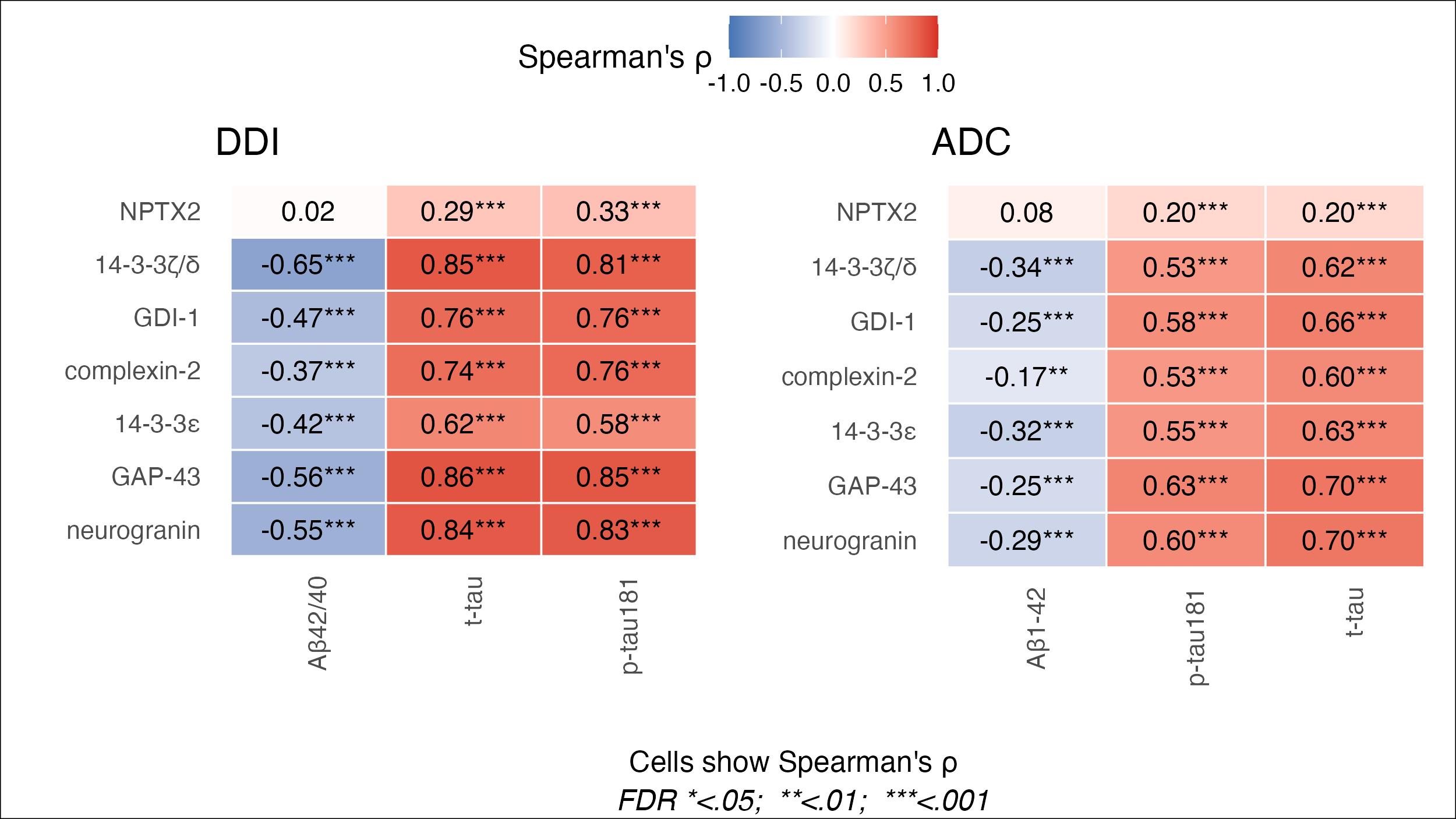


**Supplementary figure 1. Spearman’s roh correlations between core CSF Alzheimer’s Disease biomarkers with synaptic markers**. The heatmap displays Spearman’s roh (ρ) values (red = positive; blue = negative) for both cohorts: Dementia Disease Initiation (DDI) and Amsterdam Dementia Cohort (ADC). Analyses were restricted to synaptic proteins retained by the funneling procedure. Significance is indicated as * p < 0.05, ** p < 0.01, *** p < 0.001; unmarked cells are not significant.
