# Supplementary material for: Cerebrospinal fluid markers link to synaptic plasticity responses and Alzheimer’s disease genetic pathways
Source: Mol Neurodegener. 2025 Oct 13;20:107. doi: 10.1186/s13024-025-00899-w (PMC12519626; doi:10.1186/s13024-025-00899-w)
Supplement: Supplementary file 2 — Supplementary Material 2: Supplementary table 1A and 1B. Description of data: Supplementary table 1A: LC-MS/MS settings for the analysis of the synaptic and lysosomal protein panel. Supplementary table 1B: LC-MS/MS concentrations of internal standard for each peptide as well as repeatability and intermediate precision for the study after plate correction. [file 13024_2025_899_MOESM2_ESM.docx]

| **Supplementary Table 1A.** LC-MS/MS settings for the analysis of the synaptic and lysosomal protein panel. | | |
| --- | --- | --- |
|  | **Parameter** | **Setting** |
| **LC** | Sample injection volume | 40 µL |
|  | Flow-rate | 0.3 mL/min |
|  | Gradient | Broken; 5–20%B (25 min), 20–45%B (6,5 min) |
|  | Total cycle time | 35 min |
|  | Mobile phase A | 0.1% formic acid in water (v/v) |
|  | Mobile phase B | 0.1% formic acid/84% acetonitrile in water (v/v) |
| **Electrospray** | Mode | Positive |
|  | Gas temperature | 220 °C |
|  | Gas flow | 15 L/min |
|  | Nebulizer pressure | 40 psi |
|  | Sheath gas temperature | 200 °C |
|  | Sheath gas flow | 11 L/min |
|  | Capillary voltage | 3500 V |
|  | Nozzle voltage | 500 V |
| **iFunnel** | Mode | Positive |
|  | High-pressure radio frequency | 200 V |
|  | Low-pressure radio frequency | 160 V |
| **MRM method** | Retention time window | 1 min |
|  | Collision energies | Individually optimized per transition |
|  | Cell accelerator voltage | Individually optimized per transition |

| **Supplementary Table 1B**. Concentrations of internal standard for each peptide as well as repeatability and intermediate precision for the study after plate correction. | | | | |
| --- | --- | --- | --- | --- |
| Targets | |  | QC^1^ | |
|  |  | IS Conc [fmol/microL] | **Repeatability** | **Intermediate precision** |
| Protein | **Peptide** |  | **%CV^2^** | **%CV** |
| 1433E | **IISSIEQK** | **0.26** | 6.46 | 13.06 |
| 1433Z | **VVSSIEQK** | **0.26** | 2.90 | 6.15 |
| AP2B1 | **AVWLPAVK** | **0.26** | 3.31 | 5.93 |
| AP2B1 | IQPGNPNYTLSLK | **0.82** | 7.59 | 10.85 |
| CMGA | EDSLEAGLPLQVR | **4.53** | 2.31 | 7.11 |
| CMGA | **GLSAEPGWQAK** | **5.9** | 3.58 | 5.96 |
| CPLX2 | **AALEQPCEGSLTRPK** | **0.26** | 5.57 | 10.63 |
| GDIA | **QLICDPSYIPDR** | **2.6** | 8.14 | 11.57 |
| NEUG | **KGPGPGGPGGAGVAR** | **0.26** | 6.37 | 8.43 |
| NPTX1 | CESQSTLDPGAGEAR | **2.6** | 4.50 | 7.87 |
| NPTX1 | ETVLQQK | **2.6** | 2.35 | 6.62 |
| NPTX1 | **LTPGEVYNLATCSTK** | **2.6** | 2.45 | 6.98 |
| NPTX2 | ETVVQQK | **2.6** | 6.59 | 12.86 |
| NPTX2 | **VAELEDEK** | **0.26** | 3.50 | 6.45 |
| NPTXR | LVEAFGGATK | **2.6** | 4.87 | 9.99 |
| NPTXR | **NNYMYAR** | **2.6** | 1.95 | 4.35 |
| PEBP1 | LYEQLSGK | **0.26** | 2.51 | 4.89 |
| PEBP1 | **NRPTSISWDGLDSGK** | **2.6** | 1.96 | 4.71 |
| SAP3 | EVAGLWIK | **9.93** | 5.02 | 7.08 |
| SAP3 | **IESVLSSSGK** | **6.28** | 2.44 | 4.18 |
| SCG2 | ALEYIENLR | **3.45** | 4.36 | 6.70 |
| SCG2 | **VLEYLNQEK** | **7.35** | 3.52 | 5.76 |
| STX1B | **QHSAILAAPNPDEK** | **0.26** | 10.13 | 13.55 |
| STX7 | **EFGSLPTTPSEQR** | **0.26** | 4.90 | 9.79 |
| SYUB | **EGVVQGVASVAEK** | **0.26** | 11.15 | 16.25 |
| SYUG | **ENVVQSVTSVAEK** | **0.26** | 8.33 | 12.89 |
| VGF | **AYQGVAAPFPK** | **3.71** | 4.27 | 6.84 |
| VGF | NSEPQDEGELFQGVDPR | **17.1** | 6.99 | 8.14 |
| Proteins/peptides marked as bold continued with for statistical analysis. ^1^Quality control; ^2^Coefficient of variation. | | | | |
