# Supplementary material for: Cerebrospinal fluid markers link to synaptic plasticity responses and Alzheimer’s disease genetic pathways
Source: Mol Neurodegener. 2025 Oct 13;20:107. doi: 10.1186/s13024-025-00899-w (PMC12519626; doi:10.1186/s13024-025-00899-w)
Supplement: Supplementary file 6 — Supplementary Material 6: Supplementary Table 5. Description of data: Detailed statistical results from within-pathology group comparisons of synaptic marker concentrations between cognitively normal and mild cognitive impairment cases, restricted to proteins identified through our funneling approach. Analyses were conducted separately within Aβ+/Tau- and Aβ+/Tau + groups in the Dementia Disease Initiation (DDI) and Amsterdam Dementia Cohorts (ADC), with pooled effect estimates provided through meta-analyses across cohorts. [file 13024_2025_899_MOESM6_ESM.docx]

| **Supplementary table 5.** Between-group comparisons of medial temporal lobe magnetic resonance (MRI) metrics and memory recall in the Dementia Disease Initiation cohort. | | | | | |
| --- | --- | --- | --- | --- | --- |
|  | **Dementia Disease Initation Cohort**  **Aβ/Tau groups** | | | | |
|  | **CN**  **Aβ-/Tau- 154** | **CN Aβ+/Tau- 20** | **MCI Aβ+/Tau- 25** | **CN Aβ+/Tau+ 40** | **MCI Aβ+/Tau+ 107** |
| ^a^**Anterior Hippocampal volume** Mean (SD) [n] | 1755.0 (239.7)  [82] | 1594.2 (223.7)  [8] | 1542.9 (217.6)  [13] | 1790.2  (239.2) [15] | 1594.9*****  (326.5) [62] |
| ^a^**Posterior Hippocampal volume** Mean (SD) [n] | 1681.9  (161.6)  [82] | 1570.1 (141.2) [8] | 1507.3***** (167.12) [13] | 1621.0 (183.2) [15] | 1470.4******* (188.9) [62] |
| ^a^**Entorhinal Cortex** Mean (SD) [n] | 614.1  (90.6) [82] | 524.3 (50.4)  [8] | 522.7***** (103.0) [13] | 623.5 (79.5) [15] | 534.0******* (93.8) [62] |
| **CERAD memory recall** Mean (SD) | 7.6 (1.6) | 7.4 (1.6) | 3.5******* (2.7) | 6.9 (2.0) | 2.6******* (2.2) |
| Abbreviations: Aβ +/-, positive or negative CSF marker for Aß plaques (Aß42/40 ratio); Tau +/-, positive or negative marker for either CSF p-tau181 and/or total-tau; SD, standard deviation; n, number of cases; CN, Cognitively Normal; MCI, Mild Cognitive Impairment; ^a^, Subsample with available MRI scans ^;^;*, <.05,**, <.01, ***<.001 (compared to the CN Aβ-/Tau- group) | | | | | |
